# Engineering Immunoregenerative Therapy via an Immunomodulatory Binary Pharmacology Hydrogel Depot for Prolonged Allograft Survival

**DOI:** 10.1002/advs.202520994

**Published:** 2026-07-07

**Authors:** Ning Wang, Ruiqi Sun, Yang Fu, Zhonghan Wu, Xinyu Tong, Hong Tang, Wentao Zhao, Zhi Liang, Jintao Zheng, Yanan Guan, Ke Zhou, Penghong Song, Shusen Zheng, Hangxiang Wang, Haiyang Xie

**Affiliations:** ^1^ Division of Hepatobiliary and Pancreatic Surgery Department of Surgery The First Affiliated Hospital Zhejiang University School of Medicine Hangzhou China; ^2^ NHC Key Laboratory of Combined Multi‐Organ Transplantation Key Laboratory of Organ Transplantation Zhejiang Province. Institute of Organ Transplantation National Clinical Research Center for Infectious Diseases Zhejiang University. State Key Laboratory for Diagnosis and Treatment of Infectious Diseases Hangzhou China; ^3^ Division of Lung Transplantation and Thoracic Surgery Department of Surgery The First Affiliated Hospital Zhejiang University School of Medicine Hangzhou China

**Keywords:** immunoregenerative effects, immunosuppressants, organ transplantation, prodrug engineering, syringeable hydrogel

## Abstract

Immunosuppressive therapy following organ transplantation is essential for ensuring long‐term graft survival but leaves patients vulnerable to complications such as infection, malignancy, and severe side effects. Immunosuppressants currently in use are typically hydrophobic with low oral bioavailability and must be taken indefinitely to maintain immune tolerance. Furthermore, these drugs lack tissue repair capacity, which limits their effectiveness in transplantation. To address this clinical gap, an immunomodulatory hydrogel (iGEL) was developed, integrating prodrug engineering with inflammation‐restricted pharmacokinetics. A binary pharmacology‐loaded iGEL spontaneously formed upon subcutaneous implantation using a dual‐syringe system. Acting as a tissue‐adhesive depot, iGEL released key antirejection and tissue‐regenerative agents, enabling localized immunomodulation in response to rejection‐induced inflammation. In mouse major histocompatibility complex–mismatched allo‐transplant models, iGEL suppressed T‐cell activity while promoting vascular reconstruction and regulating local cytokine profiles to remodel immune‐regenerative dynamics. For both immunocompetent and metabolically compromised hosts, iGEL effectively restored the functionality of skin allografts and markedly extended survival. The study introduces a locally syringeable and adaptive hydrogel depot that harnesses pathological cues to mediate complementary immune regulation and tissue repair, ensuring long‐term graft survival without systemic immunosuppression.

## Graphical abstract

An immunomodulatory hydrogel (iGEL) forms spontaneously upon subcutaneous injection, acting as a tissue‐adhesive depot. It releases anti‐rejection and regenerative agents in response to inflammation, suppressing T‐cell activity, promoting vascular repair, and restoring allograft function without systemic immunosuppression.

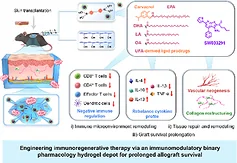

## 1 Introduction

Organ transplantation has emerged as a life‐saving therapeutic option for patients with end‐stage organ failure [1, 2, 3]. Following transplant surgery, immunosuppressive therapy must be continued for a long period to prevent allograft rejection [4, 5]. The current clinically available transplant drugs primarily comprise naturally derived and synthetic agents, most of which are small‐molecule hydrophobic compounds [6, 7]. While these agents have contributed to successful transplant outcomes, their clinical effectiveness is limited by complications such as suboptimal bioavailability, de novo malignancy, and dose‐limiting toxicities [7, 8, 9]. Moreover, existing post‐transplant management strategies fail to include regenerative support, which may increase the risk of early allograft dysfunction and failure [10, 11]. Thus, there remains a clinical need for immunosuppressive therapy that is both effective and capable of aligning with transplant regeneration, with potential applications in organ transplantation and other clinical contexts.

Conventional immunosuppressive agents (ISAs) such as tacrolimus, sirolimus, mycophenolate mofetil, and cyclosporin A typically require high doses and frequent administration to achieve immune hyporesponsiveness [5, 7, 12]. However, long‐term ISA use following transplantation weakens the recipient's immune system, raising the risk of infection, malignancy, myelosuppression, and chronic immune dysregulation [13, 14, 15, 16]. Long‐acting drug delivery systems have emerged as promising alternatives, sustaining therapeutic concentrations in vivo while reducing dosing frequency [17, 18, 19]. Among these, injectable hydrogels are particularly suitable for use in transplantation [20, 21]. They can form tissue‐adherent, biocompatible depots at graft sites, allowing for localized immunomodulation and controlled drug release [20, 21]. However, the clinical application of hydrogel‐based ISA delivery is challenging because the inherent hydrophobicity of most immunosuppressants results in poor drug loading and suboptimal dispersion within hydrophilic polymer networks [22]. Therefore, the development of long‐acting modalities that ensure sustained release in response to transplantation‐associated inflammatory cues is expected to improve clinical outcomes.

Herein, we present an immunomodulatory binary pharmacology hydrogel (iGEL) platform that integrates prodrug technology with inflammation‐restricted pharmacology to remodel immunoregenerative dynamics in transplantation. Building on our previous findings, which identified carvacrol as a natural ISA [23], we developed a chemical strategy to generate a small set of carvacrol prodrugs for subsequent supramolecular nanoassembly. The hydrophobic core of the assemblies allowed the encapsulation of the regenerative agent SW033291 [24], which was further refined by an amphiphilic polyethylene glycol–polycaprolactone (PEG–PCL) copolymer on the nanoparticle surface [25]. To avoid the “always‐on” systemic exposure that reduces efficacy and to enhance local skin allograft targeting, we designed the binary pharmacology nanoparticles into an inflammation‐responsive iGEL scaffold [26]. Upon subcutaneous implantation using a dual‐syringe system, the binary pharmacology‐loaded iGEL formed spontaneously, enabling prolonged therapeutic retention at the skin allograft site while minimizing premature leakage. We hypothesized that iGEL could create a localized immunosuppressive and regenerative microenvironment to improve skin allograft survival. By reducing T‐cell infiltration, rebalancing local cytokine profiles, and supporting vascular remodeling, iGEL substantially improved skin allograft survival in murine allogeneic skin transplantation models, including immunocompromised and metabolically dysfunctional diabetic recipients. Overall, this prodrug‐integrated hydrogel platform offers a localized and adaptive strategy for enhancing transplant durability.

## 2 Materials and Methods

### 2.1 Animals

C57BL/6 (6‐week‐old) and BALB/c (6‐week‐old) mice were obtained from Hangzhou Medical College. All mice were housed at the experimental animal center of the First Affiliated Hospital, Zhejiang University School of Medicine, with access to a standard diet and sterilized water. All animal experiments were conducted following protocols approved by the Animal Ethics Committee of Zhejiang University School of Medicine (2023‐804).

### 2.2 Screening of Molecules for Enhanced Regenerative Repair

Cell proliferation was assessed using the Cell Counting Kit‐8 assay (HY‐K0301, MCE). NIH‐3T3 cells were first seeded into 96‐well plates at a density of 3000 cells per well. After 24 h of incubation at 37°C, various concentrations of SW033291, CCT007093, ISX‐9, D‐Panthenol, TCPOBOP, TDI‐011536, FPH1, and Alisol B‐acetate (all purchased from MCE), ranging from 20 nm to 2 µm, were added to the wells. After another 48 h incubation, the culture medium was aspirated, and fresh medium containing 10% CCK‐8 reagent was added. The plates were incubated for 1 h, after which absorbance at 450 nm was measured for each well. The relative proliferation rate of NIH‐3T3 cells under each treatment was calculated against the untreated control group, following the manufacturer's instructions. After 24 h of NIH‐3T3 cell incubation, the cultures were treated with 200 nm small‐molecule compounds for 48 h. Total RNA was subsequently isolated using an RNA extraction kit (RK30120, ABclonal) as per the manufacturer's protocol, followed by reverse transcription into complementary DNA (RK20433, ABclonal). The relative mRNA expression levels of PCNA, STAT3, Cyclin A2, and Cyclin D1 were quantified via quantitative real‐time PCR (RK21203, ABclonal), with GAPDH serving as the endogenous control. The gene‐specific primer sequences are provided in Table S1.

### 2.3 Preparation of Nanoparticles

Four UFAs (oleic acid/OA, linoleic acid/LA, docosahexaenoic acid/DHA, and eicosapentaenoic acid/EPA—all purchased from MCE) were covalently conjugated with carvacrol (HY‐N0711, MCE) to create amphiphilic prodrugs (designated as Car‐OA/LA/DHA/EPA, e.g., Car‐OA: carvacrol‐oleic acid prodrug). The synthesis procedure is outlined in the Supporting Information, and successful synthesis was confirmed via ^1^H nuclear magnetic resonance (NMR). These prodrugs were then loaded into amphiphilic PEG_10k_‐*b*‐PCL_10k_ polymeric micelles (Yare, 673410k‐72510k, 19 times the total mass of carvacrol), resulting in four nanoparticles (termed CE/CD/CO/CLNP, e.g., CENP: Car‐EPA nanoparticle).

Additionally, a predetermined amount of Car‐EPA (carvacrol‐eicosapentaenoic acid prodrug), SW033291 (HY‐16968, MCE), and PEG_10k_‐*b*‐PCL_10k_ (19 times the total mass of carvacrol and SW033291) was accurately weighed and dissolved in acetone. At ambient temperature, the mixture was slowly added dropwise to deionized water with continuous stirring. The mixture was then stirred at 50°C for 30 min to completely remove acetone, producing the binary pharmacological nanoparticles (termed CSNP). Nanomaterial morphology, size distribution, and dispersity were characterized using transmission electron microscopy (TEM, Talos 120 kV, ThermoFisher) and dynamic light scattering (DLS, Malvern Panalytcal).

### 2.4 Preparation of Immunomodulatory Binary Pharmacology Hydrogel

To prepare the hydrogel, 250 mg of polyvinyl alcohol (PVA, molecular weight 47 000) was added to 10 mL of deionized water and stirred continuously at 90°C until a clear solution was obtained. The reactive oxygen species (ROS)‐responsive crosslinker (TSPBA, synthesis procedure provided in the Supporting Information) was dissolved in a suspension containing CSNP. Using a dual‐barrel syringe, the PVA solution (2.5 wt.%) and TSPBA solution (10 wt.%) were injected into the mixture simultaneously, causing the inflammation‐responsive iGEL to form instantly upon contact. Scanning electron microscopy (SEM, Nova Nano 450, Thermo FEI) was used to visualize the porous architecture of iGEL. Before SEM imaging, iGEL was rapidly frozen in liquid nitrogen and lyophilized for 12 h to induce fracturing, resulting in rough cross‐sections. Additionally, a Blank‐Gel was prepared by dissolving TSPBA in deionized water, which led to gelation upon immediate contact with PVA.

### 2.5 In Vitro Activation and Proliferation of T Cells

Murine spleen–derived lymphocytes (MSDLs) were isolated using a lymphocyte separation medium (P8860, Solarbio). The cells were plated and exposed to CENP (10 µm Car‐EPA), SW033291 (2.5 µm), or CSNP (10 µm Car‐EPA plus 2.5 µm SW033291), together with IL‐2 (2 ng/mL, RP01384, ABclonal), anti‐CD3 antibody (100340, BioLegend), and anti‐CD28 antibody (102116, BioLegend) to induce T‐cell activation and proliferation. Following a 24 h incubation, the cells were stained with Fixable Viability Stain 440UV (566332, BD Biosciences) for 15 min at room temperature, rinsed in PBS, and surface‐labeled with APC‐Cy7 anti‐mouse CD45 (557659, BD Biosciences), BUV395 anti‐mouse CD3e (565992, BD Biosciences), BUV805 anti‐mouse CD4 (741943, BD Biosciences), PE‐Cy5 anti‐mouse CD8a (561094, BD Biosciences), BUV661 anti‐mouse CD44 (741471, BD Biosciences), and BUV737 anti‐mouse CD62L (612833, BD Biosciences) antibodies at 4°C in the dark for 30 min. To detect perforin and granzyme B, the lymphocytes were stimulated using a cell activation cocktail (423304, BioLegend) for 4 h, fixed and permeabilized using the Cyto‐fast fix/perm buffer kit (426803, BioLegend), and stained intracellularly with PE‐Cy7 anti‐mouse granzyme B (372214, BioLegend) and APC anti‐mouse perforin (154304, BioLegend). Data were acquired using a Cytek Aurora full‐spectrum flow cytometer.

To evaluate T‐cell proliferation, MSDLs were labeled with carboxyfluorescein succinimidyl ester (CFSE, C34570, Invitrogen) and cultured with IL‐2, anti‐CD3 antibody, and anti‐CD28 antibody. Following a 5‐day incubation with the drugs, MSDLs were stained with APC anti‐mouse CD4 antibody (100412, BioLegend) and PE‐Cy7 anti‐mouse CD8a antibody (561097, BD Biosciences). Flow cytometry was then performed to measure CFSE dilution in T lymphocytes as an indicator of cell proliferation.

### 2.6 Evaluation of Inflammation‐Responsive Release Kinetics

High‐performance liquid chromatography (HPLC) was used to evaluate inflammation‐responsive drug release. Specifically, 2 mL of iGEL (containing 0.375 mg equivalents of carvacrol and 0.125 mg SW033291) was sealed in dialysis bags with a molecular weight cut‐off of 7000 Da. The dialysis bags were individually immersed in three different release media (each with 0.4% Tween‐80): PBS, 1 mM hydrogen peroxide (H_2_O_2_), or 10 mm H_2_O_2_. The experiments were conducted in a 37°C shaking incubator. At scheduled intervals, external media were collected and replaced with equal volumes of fresh solution. The collected media were treated with 0.1 m NaOH and incubated at 37°C for 4 h, followed by neutralization with 0.1 m HCl. The samples were subsequently analyzed using HPLC to generate release curves under the three different release conditions.

### 2.7 Murine Skin Transplantation Model

Six‐week‐old male C57BL/6 mice (recipients) underwent dorsal skin preparation followed by excision of 1 × 1 cm^2^ full‐thickness skin to create recipient beds. Identical‐size grafts were obtained from age‐matched BALB/c mice (donors); these skin allografts were affixed to recipient beds using 6–0 sutures and covered with dressings. To establish a type I diabetic transplantation model, recipient C57BL/6 mice were intraperitoneally injected with streptozotocin (70 mg/kg/day, S8050, Solarbio) for 5 consecutive days [27]. Fasting blood glucose levels were monitored weekly, and diabetes was diagnosed when two successive measurements exceeded 11.1 mmol/L, accompanied by polyuria and polydipsia. Skin allografts from age‐matched BALB/c donors were transplanted onto diabetic recipients using the same surgical procedure described above.

Following successful establishment of the transplant model, the mice were randomly divided into five treatment groups: (1) Saline: intravenous injection of saline; (2) Free Drug (FD): intraperitoneal injection of carvacrol (30 mg/kg) plus SW033291 (10 mg/kg); (3) CSNP: intravenous injection of nanoparticles containing Car‐EPA (molar‐equivalent dose of 30 mg/kg carvacrol) and SW033291 (10 mg/kg); (4) Blank‐Gel: peri‐transplant implantation of unloaded hydrogel scaffold; and (5) iGEL: peri‐transplant implantation of CSNP‐loaded hydrogel. Starting from postoperative day 7 (POD7), daily digital imaging was performed to monitor skin allograft progression until full detachment occurred, and skin allograft survival scores (SGSSs) were recorded according to the necrotic area percentage, with higher necrosis yielding lower SGSSs (0 points at complete detachment) [23].

### 2.8 Local Accumulation of Carvacrol and SW033291

For biodistribution studies, the mice with established skin allografts were randomly assigned to three groups: (1) Free DiR: intravenous injection of free 1,1'‐dioctadecyl‐3,3,3',3'‐tetramethylindotricarbocyanine iodide (DiR, 1 mg/kg, abs45153692, Absin); (2) CSNP^DiR^: intravenous injection of DiR‐loaded CSNP (1 mg/kg DiR, 10 mg/kg SW033291, and 30 mg/kg molar‐equivalent of carvacrol); (3) iGEL^DiR^: peri‐transplant implantation of hydrogel scaffolds preloaded with DiR‐containing CSNP. At scheduled intervals, in vivo distribution was evaluated using a small animal imaging system (IVIS Lumina LT, Revvity), followed by ex vivo fluorescence measurement of skin allografts, graft‐draining lymph nodes (gdLNs), and major organs (heart, liver, spleen, lungs, and kidneys).

Drug levels in the skin allografts and gdLNs were quantified using HPLC. Mice bearing skin allografts were randomized into three groups: FD (10 mg/kg SW033291 and 30 mg/kg carvacrol), CSNP (10 mg/kg SW033291 plus 30 mg/kg molar‐equivalent of carvacrol), and iGEL. At scheduled time points, the skin allografts and gdLNs were harvested, weighed, and homogenized. The tissue samples were incubated in 0.1 M NaOH at 37°C for 4 h, neutralized with 0.1 m HCl, and analyzed spectroscopically at 280 nm.

### 2.9 Detection of ROS Level

To explore the inflammation‐responsive release mechanism at physiologically and pathologically relevant ROS concentrations, allogeneic (Balb/c (H‐2d) to C57BL/6 (H‐2b)) and syngeneic (C57BL/6 (H‐2b) to C57BL/6 (H‐2b)) skin transplantation models in mice were constructed. On post‐transplantation days 4 (POD4) and 8 (POD8), skin grafts and axillary lymph nodes were collected. Skin tissues were minced and digested with collagenase I (2 mg/mL, CC3781G, Coolaber) at 37°C for 2 h, followed by trypsin digestion for 10 min. Relative intracellular oxidative status was assessed using a DCFH‐DA‐based reactive oxygen species detection kit (S0034M, Beyotime). To explore the iGEL's responsiveness to physiological and pathological ROS levels, both allogeneic and syngeneic skin transplantation models were conducted. iGEL loaded with DiR dye was administered peri‐transplant, and in vivo fluorescence imaging was performed at 48 and 96 h post‐treatment.

### 2.10 Flow Cytometry Analysis

On POD9, the spleen, gdLNs, and peripheral blood were harvested from each treatment group. Following mechanical dissociation via gentle grinding, single‐cell suspensions were prepared from the spleen and gdLNs, and red blood cells (RBCs) were removed using lysis buffer (R1010, Solarbio). Each suspension was then split into two fractions for separate analysis of the lymphoid and myeloid subsets. For lymphoid cell profiling, the cells were stained with Zombie NIR (423105, BioLegend), FITC anti‐mouse CD45 antibody (561088, BD Biosciences), BV510 anti‐mouse CD3e antibody (563024, BD Biosciences), APC‐Cy7 anti‐mouse CD4 antibody (561830, BD Biosciences), PerCP anti‐mouse CD8a antibody (561092, BD Biosciences), APC anti‐mouse NK1.1 antibody (561117, BD Biosciences), AF488 anti‐mouse Foxp3 antibody, BUV661 anti‐mouse CD44 antibody (741471, BD Biosciences), and BV605 anti‐mouse CD62L antibody (563252, BD Biosciences). For myeloid cell characterization, the cells were stained with Fixable Viability Stain 620 (564996, BD Biosciences), BV421 anti‐mouse CD11b antibody (562605, BD Biosciences), FITC anti‐mouse CD11c antibody (561045, BD Biosciences), PE‐Cy7 anti‐mouse F4/80 antibody (123114, BioLegend), PE anti‐mouse MHC‐II antibody (562010, BD Biosciences), PE‐CF594 anti‐mouse CD80 antibody (562504, BD Biosciences), BV510 anti‐mouse CD86 antibody (563077, BD Biosciences), APC anti‐mouse CD206 antibody (162506, BioLegend), and PerCP‐Cy5.5 anti‐mouse Ly6G antibody (560602, BD Biosciences). Data were acquired using a Cytek Aurora full‐spectrum cytometer and processed with FlowJo v10.

### 2.11 Multiplex Cytokine Profiling

On POD9, skin allografts and peripheral blood were collected. The skin allografts were minced and homogenized to generate tissue lysates, which were centrifuged to recover the supernatant. Protein concentration was measured using a bicinchoninic acid assay (20201ES, Yeasen), and all sample concentrations were normalized to 3 mg/mL. A mouse multiplex custom panel (RK04391/RK04379, ABclonal) was then used to quantify cytokines in tissue lysates and serum. IL‐2 and CCL3 in skin allograft samples, as well as circulating IL‐2, VCAM‐1, IL‐13, and CCL4, were undetectable or below the limit of quantification. Cytokine levels were Z‐score normalized to reduce interexperiment variation. Principal component analysis (PCA) was applied for dimensionality reduction, and the results were displayed as 2D scatter plots for comparison.

### 2.12 Transcriptome Sequencing and Analysis

On POD8, skin allografts were harvested for RNA extraction. RNA quality was assessed on a 5300 Bioanalyzer (Agilent) and quantified with the ND‐2000 spectrophotometer (NanoDrop Technologies). Libraries for mRNA‐seq were prepared using Ligation from Illumina Stranded RNA Prep with 1 µg of total RNA as input. Raw reads were quality‐filtered with fastp to remove adapters and bases with Phred scores of < 20. Differential expression analysis was performed with DESeq2 using |FoldChange| > 2 and adjusted *p* < 0.05 as cutoffs. Gene Ontology (GO) enrichment was conducted using Goatools.

### 2.13 Murine Acute Wound Healing Model

Following hair removal, a circular full‐thickness wound (10 mm in diameter) was generated on the dorsum of C57BL/6 mice using a biopsy punch. The animals were then randomly allocated to seven groups: (1) Saline: intraperitoneal saline injection; (2) Carvacrol: intraperitoneal carvacrol injection; (3) SW033291: intraperitoneal SW033291 injection; (4) FD: intraperitoneal carvacrol plus SW033291 injection; (5) CSNP: intravenous Car‐EPA/SW033291 nanoparticle injection; (6) Blank‐Gel: wound bed implantation of blank hydrogel; and (7) iGEL: wound bed implantation of CSNP‐loaded hydrogel. Therapeutic doses were fixed at 30 mg/kg for carvacrol and 10 mg/kg for SW033291. Wound morphology was imaged on PODs 3, 6, and 9, and wound areas were quantified using ImageJ to calculate closure rates. On POD9, skin tissues from the wound region were collected for hematoxylin‐eosin (H&E) staining and immunohistochemistry (IHC).

### 2.14 Histopathological Analysis

Collected tissues were fixed in 4% paraformaldehyde, embedded in paraffin, and sectioned. H&E staining was performed to assess the skin allograft structure and the newly formed granulation tissue. IHC staining for CD31 (ab182981, Abcam) and Cyclin D1 (55506S, CST) was performed to evaluate vascularization and proliferative activity, respectively. Lymphocyte infiltration and collagen deposition in the skin allografts were examined via CD3 (ab11089, Abcam) immunostaining and Masson's trichrome staining. Apoptosis was detected using a TUNEL Apoptosis Detection Kit (FITC, 40306ES, Yeasen; Alexa Fluor 594, C1176S, Beyotime). For analysis of T‐cell subsets, dual immunofluorescence staining was performed using mouse monoclonal anti‐CD4 (67786‐1, Proteintech) and rabbit monoclonal anti‐CD8a (ab237723, Abcam) primary antibodies, followed by Alexa Fluor 488–conjugated anti‐mouse IgG (H+L) F(ab')2 (4408, CST) and Alexa Fluor 594–conjugated anti‐rabbit IgG (H+L) F(ab')2 (8889, CST). Nuclei were counterstained with DAPI (P0131, Beyotime).

### 2.15 Biosafety Evaluation

Six‐week‐old healthy C57BL/6 mice were further divided into the FD, CSNP, or iGEL treatment groups and were administered once every 3 days for a total of three doses. Two regimens were used: low dose (30 mg/kg carvacrol, 10 mg/kg SW033291) and high dose (45 mg/kg carvacrol, 15 mg/kg SW033291). Body weight was monitored throughout as a general health indicator. Three days after the final treatment, blood was collected for complete blood count and liver/kidney function assays. Major organs (heart, liver, spleen, lungs, and kidneys) were harvested for detailed histopathological evaluation. In addition, blood samples were collected at 30 and 50 days post‐iGEL treatment for the determination of liver and kidney functions. Histopathological examinations were conducted on the skin tissues at the hydrogel deposition sites in the iGEL group. These examinations were carried out at 10, 15, 20, 30, and 50 days after the completion of three hydrogel injections to evaluate the extent of local tissue damage. Additionally, to evaluate the safety of iGEL under transplantation‐associated inflammatory conditions, we applied the same treatment regimens (low dose: 30 mg/kg carvacrol and 10 mg/kg SW033291; high dose: 45 mg/kg carvacrol and 15 mg/kg SW033291) in an allogeneic skin transplantation model. Liver and kidney functions were assessed at two time points: 10 days post‐transplantation, when allografts were still present, and 30 days post‐transplantation, after allograft loss. These measurements were compared with age‐matched healthy C57BL/6 mice to determine potential systemic effects of iGEL in the context of alloimmune inflammation.

### 2.16 Statistical Analysis

All data are expressed as mean ± standard deviation (SD). Statistical analyses were conducted in GraphPad Prism 9 using one‐way ANOVA followed by Tukey's post hoc test and two‐way ANOVA followed by Tukey's posthoc or log‐rank (Mantel−Cox) test. A *p*‐value < 0.05 was considered statistically significant.

## 3 Results

### 3.1 Development of the Binary Nanoparticle Pharmacology‐Incorporated ROS‐Responsive iGEL Platform

Our previous studies showed that upon aqueous self‐assembly, UFA‐derived lipid prodrugs can spontaneously form hydrophobic cores, enabling the noncovalent encapsulation of additional water‐insoluble therapeutics [8]. Based on this supramolecular approach, we aimed to synthesize UFA‐conjugated immunosuppressive derivatives to test their self‐assembling properties (Figure 1). The hydrophobic core of these nanoassemblies could support the coencapsulation of proregenerative agents, resulting in binary pharmacology nanoparticle systems. Additionally, these binary nanoparticles were incorporated into a polyvinyl alcohol (PVA)‐based hydrogel matrix connected by a ROS‐responsive linker (TSPBA, Figure S1). Four carvacrol derivatives conjugated with either polyunsaturated fatty acids (e.g., DHA, EPA, LA) or a monounsaturated fatty acid (e.g., OA), were synthesized and characterized using ^1^H NMR spectroscopy (Figures S2–S5). These compounds demonstrated the ability to replicate self‐assembly behavior in aqueous media independent of external excipients. PEGylation through amphiphilic PEG‐*b*‐PCL matrices further reduced surface hydrophobicity, improving colloidal stability [22]. TEM images confirmed the spherical structure of the nanoparticles (Figure S6). The particle size was measured via DLS analysis, ranging from ∼90 to ∼150 nm, with a narrow monomodal distribution (Figure S6). Among these lipid nanoassemblies, the nanoparticles derived from the Car‐EPA prodrug (termed CENP) was identified as the top candidate, offering favorable stability for long‐term storage (Figure S6), superior T cell proliferation inhibition in vitro (Figure S7), and extended skin allograft survival in vivo (Figure S8).

**FIGURE 1:**
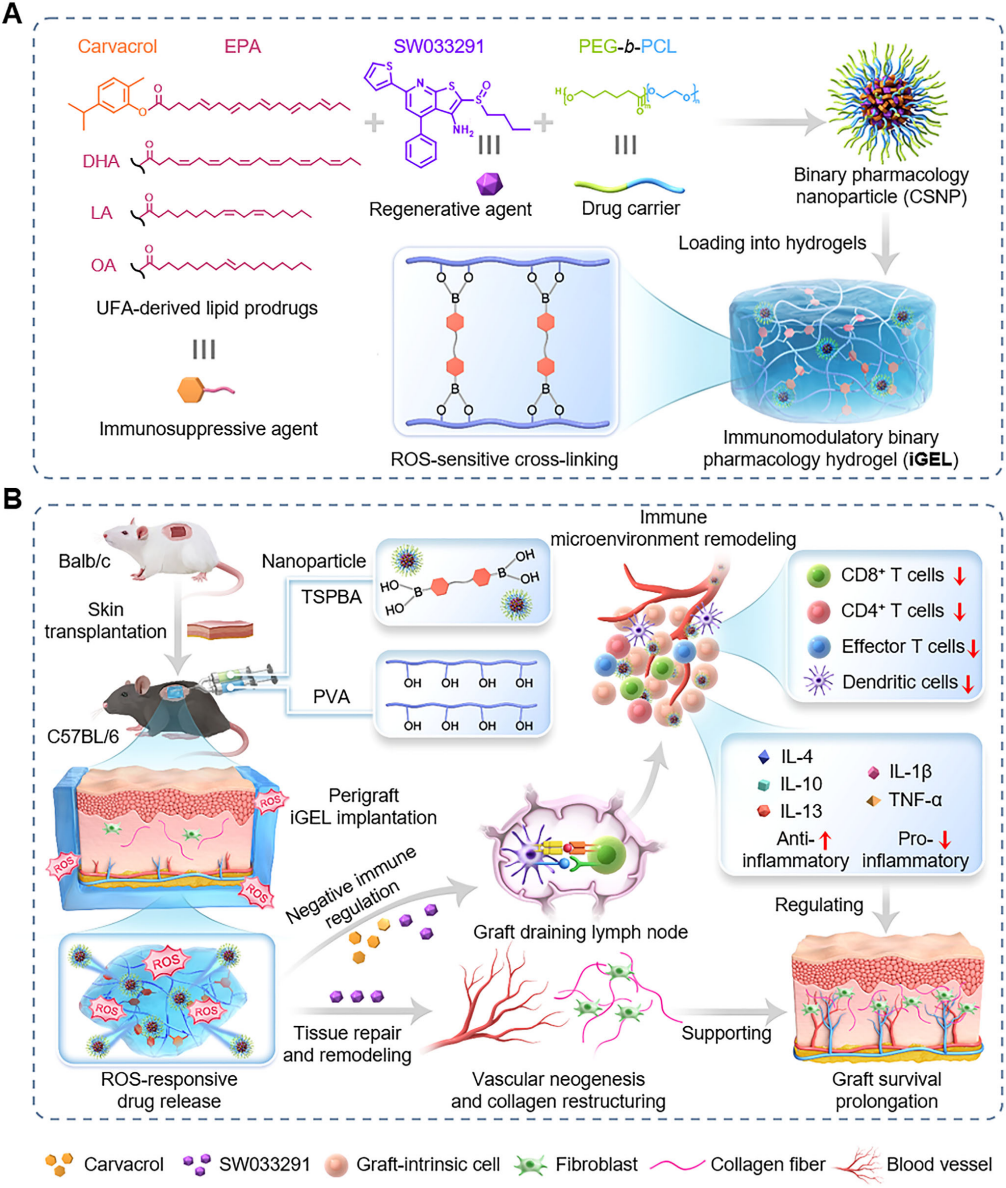
Schematic illustration of ROS‐responsive iGEL codelivering carvacrol and SW033291 to mitigate skin allograft rejection. (A) Fabrication of an injectable dual‐shield hydrogel scaffold. (B) ROS‐triggered hydrogel degradation at the transplant site initiates the synchronized release of carvacrol and SW033291, which modulating inflammatory homeostasis in the immune microenvironment while promoting angiogenesis and ordered collagen deposition, thereby mediating complementary immunoregulatory and regenerative effects to reduce skin allograft rejection.

In this construct, the *π‐*rich and hydrophobic inner core structure provided by the Car‐EPA conjugate was expected to accommodate additional water‐insoluble, pharmacologically active compounds. To create a binary pharmacology for synchronizing immunosuppression and tissue regeneration, we conducted a fibroblast‐based screening of a small panel of proproliferative compounds. This led to the identification of the small molecule SW033291 as the potent proregenerative candidate (Figure 2A and Figure S9). Remarkably, SW033291 was easily integrated into the core structure formed by the Car‐EPA conjugate, resulting in a binary pharmacology nanoparticle (termed CSNP) with similar spherical morphology and colloidal stability (Figure 2B–D). Approximately 99% of carvacrol and 80% of SW033291 were stably entrapped in CSNP, with negligible leakage observed over seven days (Figure S10). SW033291 administration induces immune suppression by modulating CD8^+^ T‐cell responses in transplanted grafts, thereby alleviating lung allograft rejection [28]. To further evaluate potential synergistic interactions between carvacrol and SW033291, we performed in vitro combination index (CI) analyses. Although the combination exhibited a CI value below 1 for inhibition of IFN‐γ expression in CD4^+^ T cells (Figure S11A, B), no consistent synergistic effects were observed across other rejection‐related parameters, including IFN‐γ, perforin, and granzyme B expression in CD8^+^ T cells, suggesting predominantly complementary rather than synergistic interactions (Figure S11C–H). Consistent with their combined immunomodulatory activity, CSNP markedly decreased the frequency of effector cells (CD44^+^CD62L^−^) among both CD4^+^ and CD8^+^ T‐cell populations (Figure 2E,F). Additionally, CSNP treatment notably downregulated perforin expression and granzyme B secretion in CD8^+^ T cells (Figure 2G,H), indicating inhibition of T‐cell activation. Notably, SW033291 exhibited a context‐dependent biphasic effect on T‐cell proliferation (Figure 2I), promoting proliferative expansion under unstimulated conditions (Figure 2J) but exerting minimal impact on activated T cells (Figure 2K). Thanks to the activity of the carvacrol prodrug, CSNP effectively suppressed T‐cell proliferation regardless of stimulation status (Figure 2I–K and Figure S12). In conclusion, these data show that CSNP mediate coordinated immunosuppressive effects by concurrently inhibiting both T‐cell activation and proliferative potential in vitro.

**FIGURE 2:**
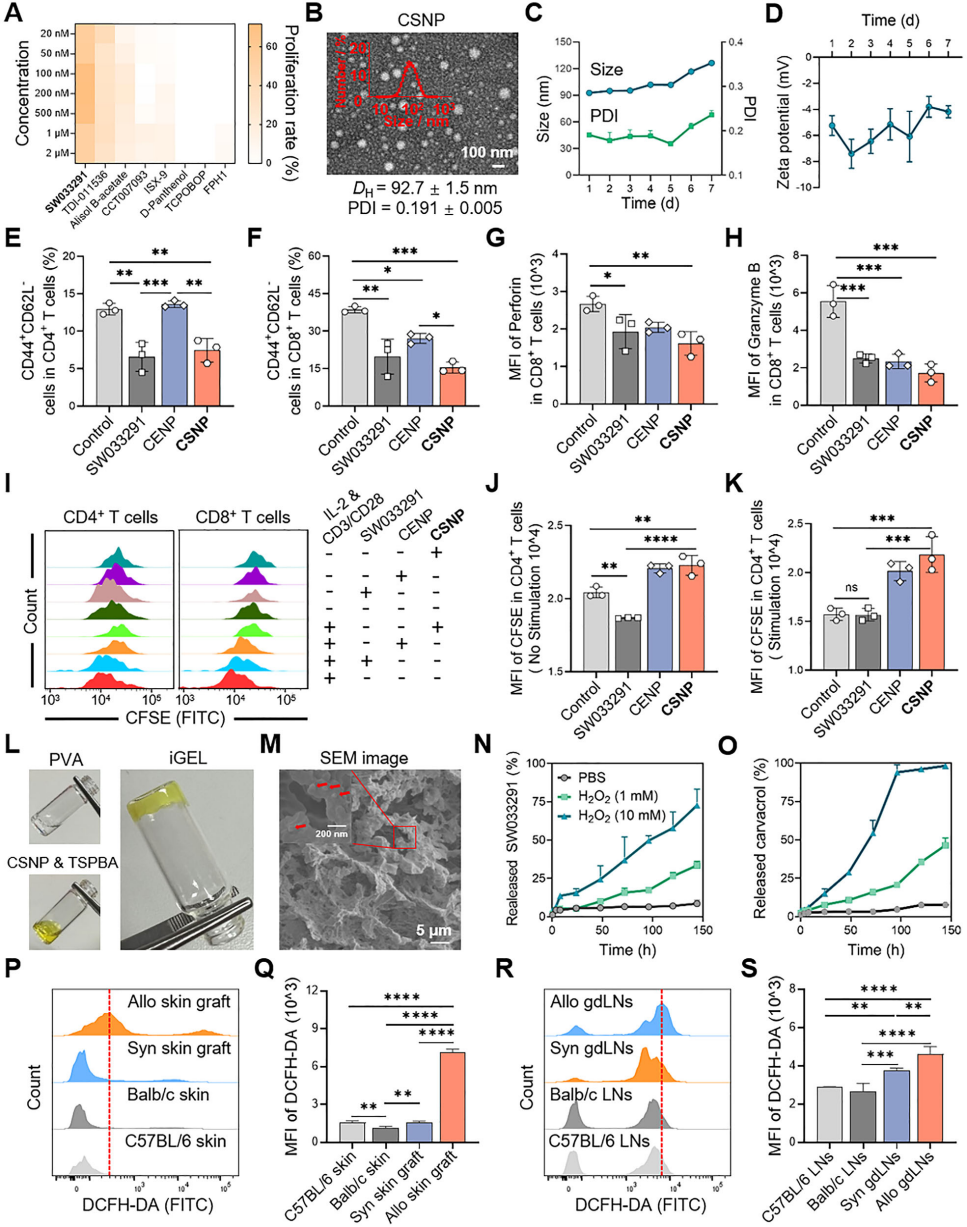
In vitro structural characterization and functional assessment of iGEL. (A) Relative cell proliferation rates of NIH‐3T3 cells treated with different regenerative small‐molecule drugs for 48 h, measured by CCK‐8 assay. (B) Representative TEM image and corresponding size distribution of CSNP. Scale bar: 100 nm. (C) Size stability and PDI of CSNP in ddH_2_O monitored by DLS at 37°C over 7 days. (D) Zeta potential changes of CSNP over 7 consecutive days. (E and F) Frequencies of CD44^+^CD62L^−^ subsets within CD4^+^ T cells and CD8^+^ T cells. (G and H) MFI of perforin and granzyme B in CD8^+^ T cells. (I) Inhibitory effects of CENP, CSNP, or SW033291 on T‐cell proliferation. (J, K) MFI of CFSE in CD4^+^ T cells under unstimulated or IL‐2/CD3/CD28‐stimulated conditions. (L) Macroscopic morphology of iGEL. (M) Representative SEM image of iGEL. Red arrows indicate attached nanoparticles. Scale bar: 5 µm or 200 nm. (N and O) Cumulative release profiles of SW033291 and carvacrol in 1 mM or 10 mM H_2_O_2_ compared with PBS control. (P) Flow cytometry histograms of DCFH‐DA fluorescence intensity in skin tissues on POD8. Allo skin graft: Skin graft in the Balb/c‐to‐C57BL/6 skin transplantation model. Syn skin graft: Skin graft in the C57BL/6‐to‐C57BL/6 skin transplantation model. (Q) Statistical analysis of the MFI of DCFH‐DA in skin tissues on POD8. (R) Flow cytometry histograms of DCFH‐DA fluorescence intensity in lymph nodes on POD8. Allo gdLNs: Graft draining lymph nodes in the Balb/c‐to‐C57BL/6 skin transplantation model. Syn gdLNs: Graft draining lymph nodes in the C57BL/6‐to‐C57BL/6 skin transplantation model. (S) Statistical analysis of the MFI of DCFH‐DA in lymph nodes on POD8. Data are expressed as mean ± SD (n = 3 in C to H, J, K, N, and O; n = 5 in P to S). Statistical significance was assessed by one‐way ANOVA with Tukey's post hoc test (E to H, J, K, Q, and S): ^*^ *p* < 0.05; ^**^ *p* < 0.01; ^***^ *p* < 0.001; ^****^ *p* < 0.0001, and ns, not significant.

For locoregional and sustained therapeutic delivery, this binary nanoparticulate platform was further encapsulated into syringeable gel matrices to form iGEL. iGEL can be easily prepared by rapidly mixing aqueous solutions of TSPBA‐containing CSNP and PVA (Figure 2L), allowing subcutaneous implantation via a dual syringe and in situ gel formation at the diseased site. SEM images revealed that iGEL had a porous structure with uniformly distributed CSNP (Figure 2M). The maximum injection force for the PVA solution was lower than 1.2 N within 4 h, while TSPBA (containing CSNP) showed a mild time‐dependent increase to ∼2.3 N at 4 h, both well below the 30 N clinical comfort threshold (Figure S13A–D). Rheological analysis confirmed stable viscosity for the PVA and TSPBA solutions at high shear rates, and successful hydrogel network formation with robust elastic properties (Figure S13E–J). iGEL was degradable in response to 10 mm H_2_O_2_, with a gradual decrease in *G'* and increase in tanδ (Figure S13G–J). Swelling assays further revealed pH‐dependent degradation, while 10 mm H_2_O_2_ induced complete dissolution within 5 days, suggesting inflammation‐derived ROS‐triggered sustainable drug release (Figure S13K). To further characterize the inflammation‐responsive degradation of iGEL, hydrogels were incubated with H_2_O_2_, and the release kinetics of SW033291 and carvacrol were monitored via HPLC (Figure 2N,O). In the absence of H_2_O_2_, Carvacrol and SW033291 were scarcely released, but both therapeutics were significantly activated upon the addition of H_2_O_2_. For example, after 96 h of incubation (10 mM H_2_O_2_), carvacrol release was quantitatively observed, while SW033291 was released at ∼50%. This difference is likely due to the distinct molecular size and retention within the hydrogels. These results highlight the stability of iGEL under physiological conditions and its responsive activation in pathologically oxidative microenvironments.

To validate our hypothesis that skin allograft rejection induces the pathological oxidative microenvironment in the GIM, which supports the iGEL's ability to release therapeutic agents, we constructed allogeneic and syngeneic skin transplantation models in mice. Compared to skin tissues from healthy C57BL/6 and Balb/c mice, as well as syngeneic grafts, allogeneic skin grafts exhibited significantly higher DCFH‐DA fluorescence intensity (Figure 2P,Q and Figure S14), indicating an elevated oxidative status in the rejection microenvironment. Additionally, DCFH‐DA MFI in the gdLNs of allogeneic grafts were also significantly higher than healthy mouse LNs (Figure 2R,S and Figure S14). In the allogeneic transplantation models, fluorescence signal intensity at the graft site was attenuated compared to normal skin, indicating the hydrogel degradation under inflammatory conditions (Figure S15). In contrast, DiR‐labeled iGEL showed more sustained fluorescence retention in normal skin for up to 96 h, suggesting slower degradation or dye release under non‐inflammatory conditions (Figure S15). Furthermore, in conditioned media from gdLN‐derived lymphocytes, iGEL cumulatively released ∼9% of carvacrol and SW033291 after 84 h incubation, which was faster than that observed in the PBS control (Figure 2N,O and Figure S16). Thus, these findings are consistent with inflammation‐promoted iGEL degradation and therapeutic release in the skin allograft microenvironment during rejection.

### 3.2 Preferential Accumulation and On‐Demand Release in Graft Immune Microenvironment (GIM)

We hypothesize that iGEL serves as a sustainable therapeutic depot, enabling long‐term release of binary pharmacology in response to the disease state. To test this hypothesis, we used a murine skin allograft model to track DiR‐labeled CSNP (hereafter referred to as CSNP^DiR^) in vivo using IVIS imaging (Figure 3A). Compared to intravenously administered CSNP^DiR^ or free DiR, iGEL^DiR^ implantation resulted in significantly higher fluorescence intensity and retention in skin allografts (Figure 3B). Notably, while CSNP^DiR^‐treated allografts showed only a transient signal increase that quickly declined and became almost undetectable by 72 h, iGEL^DiR^ maintained detectable fluorescence signals in skin allografts for up to 168 h post‐implantation. At POD8, fluorescence signals further weakened, and by POD10, signals completely disappeared (Figure S17), indicating an ∼8‐day sustained drug release period in the acute rejection microenvironment. Ex vivo imaging (Figure 3C) and quantitative analyses (Figure 3D) at predetermined time points revealed distinct distribution patterns: iGEL^DiR^ predominantly localized to skin allografts (Figure 3C,D) and gdLNs (Figure 3C,E), whereas CSNP^DiR^ primarily accumulated in highly vascularized healthy organs such as the liver, lungs, and kidneys (Figure 3F–I and Figure S18).

**FIGURE 3:**
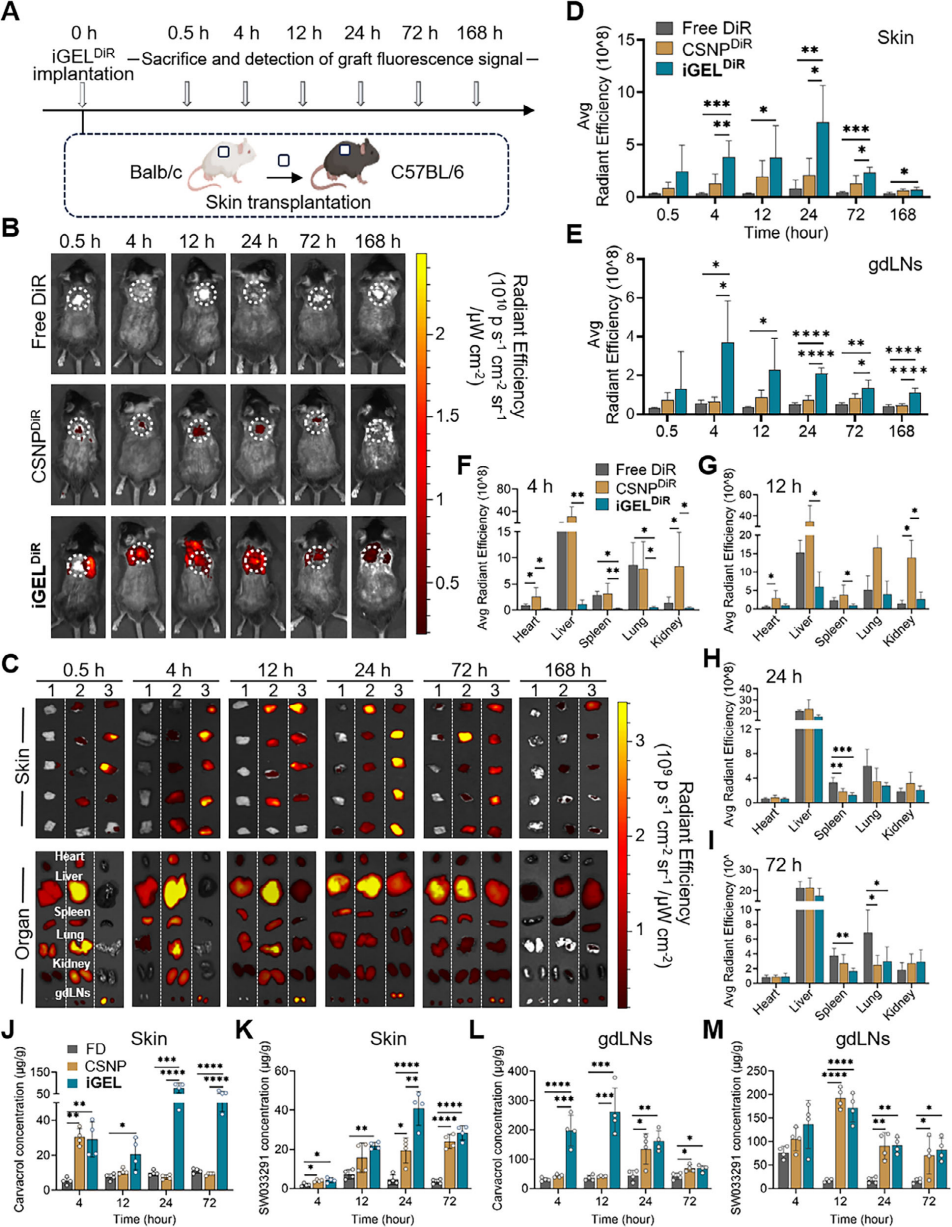
Accumulation of iGEL in the graft immune microenvironment. (A) Schematic diagram of the in vivo drug accumulation study. (B) Representative fluorescence images showing iGEL retention around the skin allografts. (C) Ex vivo fluorescence imaging of skin allografts and major organs (heart, liver, spleen, lungs, kidneys, and axillary lymph nodes). 1: Free DiR group; 2: CSNP^DiR^ group; 3: iGEL^DiR^ group. (D, E) Quantitative analysis of fluorescence signals in skin allografts and gdLNs. (F–I) Time‐course quantification of fluorescence in major organs at indicated time points. (J–M) HPLC analysis of carvacrol and SW033291 accumulation in skin allografts and gdLNs. Data are expressed as mean ± SD (n = 4–5 in D to I; n = 4 in Jto M). Statistical significance was assessed by one‐way ANOVA with Tukey's post hoc test (D toM): ^*^ *p* < 0.05; ^**^ *p* < 0.01; ^***^ *p* < 0.001; and ^****^ *p* < 0.0001.

To further quantify the drug distribution profile, we measured the levels of carvacrol and SW033291 in the skin allografts and gdLNs using HPLC analysis. iGEL treatment led to significantly higher drug concentrations in the skin allografts compared to free drug (FD) or CSNP treatment (Figure 3J,K). Specifically, carvacrol levels in the iGEL‐treated skin allografts were eight times higher than those in the FD group at 24 h and five times higher at 72 h post‐administration. Similar trends were observed in the gdLNs of iGEL‐treated mice (Figure 3L,M). UV–vis spectrophotometry analysis was employed to quantify Car‐EPA and free carvacrol in skin allografts and gdLNs. Carvacrol and Car‐EPA exhibited distinct absorption peak at 275 and 265 nm, respectively, and linear calibration curves were confirmed for both compounds (Figure S19A, B). Regardless of administration route (CSNP intravenous or iGEL local), elevated oxidative status in skin allografts and gdLNs during rejection (Figure 2P–S) may contributed to the chemical conversion of the Car‐EPA conjugate to free carvacrol (Figure S19C), supporting our allograft rejection‐restricted therapeutic activation hypothesis. Notably, iGEL demonstrated superior on‐demand release kinetics, with significantly higher carvacrol concentrations in skin allografts and gdLNs at 24 and 72 h compared to CSNP (Figure S19C). These pharmacokinetic data support the role of iGEL in enhancing therapeutic delivery to the GIM, a key immunologically relevant compartment, while minimizing the risk of systemic side effects.

### 3.3 iGEL Local Treatment Exhibits Superior Efficacy in Alleviating Skin Allograft Rejection

Allogeneic skin transplantation encounters major clinical challenges owing to severe rejection responses [20, 23]. Motivated by its strong in vitro immunosuppressive activity and enhanced drug delivery efficiency in skin allografts and gdLNs, we investigated the immunoprotective efficacy of iGEL in an allogeneic skin transplantation model by monitoring skin allograft morphology and survival (Figure 4A). iGEL was applied to the recipient's denuded graft bed, followed by the placement of full‐thickness skin allografts from the donor onto the iGEL surface and suturing. The mice treated with iGEL showed good tolerance to the procedure and maintained stable body weights throughout the study (Figure 4B). Macroscopic evaluation of the skin allografts provided a direct measure of graft viability. Skin allografts in the CSNP and FD treatment groups showed eschar formation, contracture, or sloughing within 14 days, signaling severe acute rejection (Figure 4C). Alternatively, allogeneic skin grafts treated with iGEL retained their intact morphology and showed good adherence to the wound site (Figure 4C). Consistently, iGEL treatment resulted in the highest skin allograft survival scores (SGSSs; Figure 4D) and significantly prolonged median skin allograft survival (22.7 ± 1.6 days) compared to CSNP (18.0 ± 1.8 days) and FD (16.9 ± 1.8 days) treatments (Figure 4E). This difference may be due to the inability of conventional FD (intraperitoneal) and nanocarrier (intravenous) treatments to maintain therapeutic drug levels, thus undermining the complementary benefits of the binary system. Histopathological analysis revealed that iGEL‐treated skin allografts exhibited near‐normal histological structure, minimal inflammatory cell infiltration, well‐preserved collagen fibers, active cell regeneration, and intact vascular structures (Figure 4F and Figure S20). These findings collectively suggest that iGEL provides both immunomodulatory and tissue‐regenerative effects, along with favorable in vivo tolerability, ultimately improving skin allograft survival outcomes.

**FIGURE 4:**
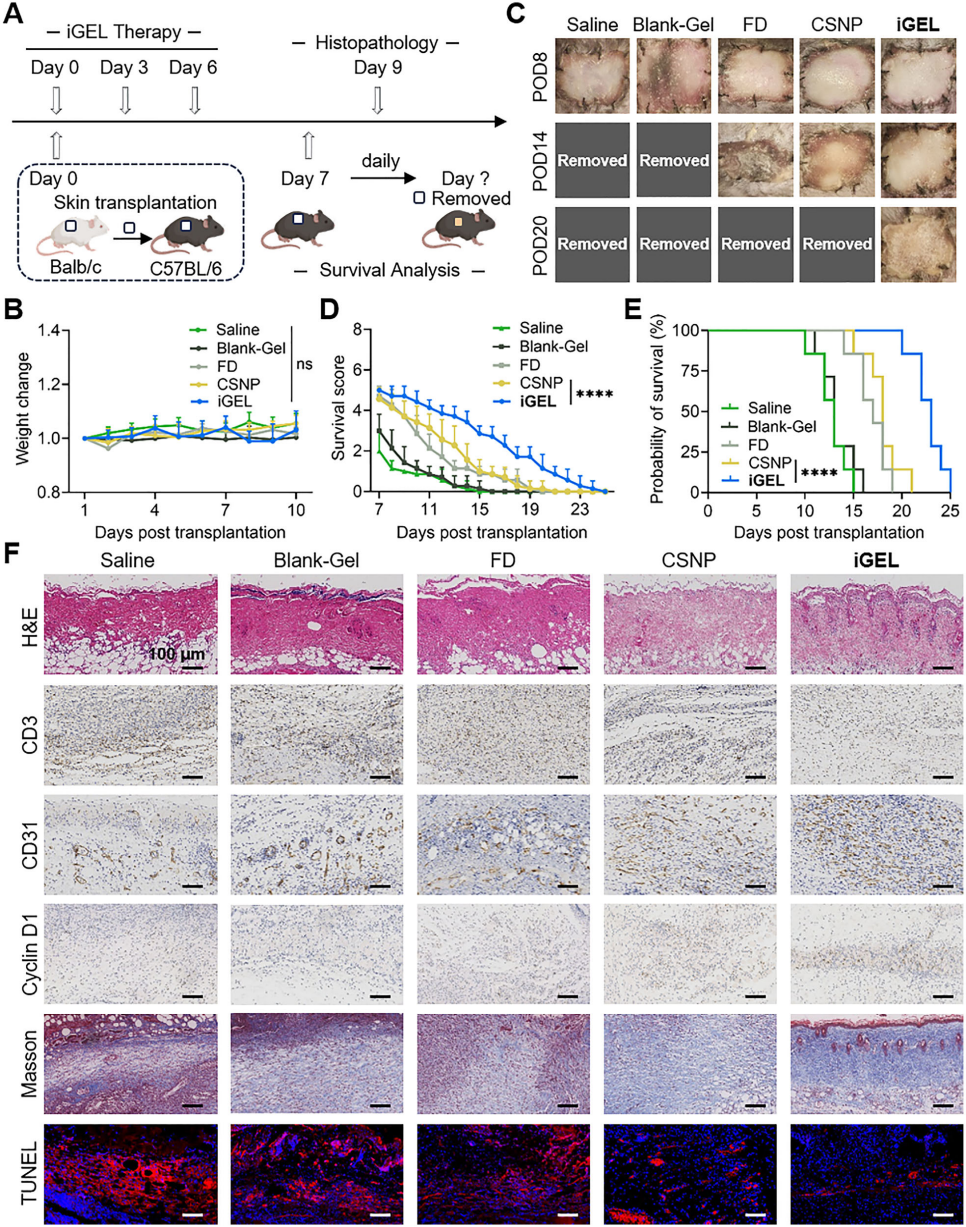
Application of iGEL in allogeneic skin transplantation. (A) Schematic of the iGEL treatment protocol and monitoring timeline after skin transplantation. (B) Body weight changes in mice during treatment. (C) Representative gross morphology of skin allografts in different treatment groups. (D) Quantitative analysis of skin allograft survival scores from POD7 until skin allograft loss. (E) Kaplan–Meier survival curves of skin allografts over 25 days post‐transplantation. (F) Histopathological evaluation on POD9: H&E staining, immunohistochemistry (CD3^+^ T cells, CD31^+^ vasculature, Cyclin D1^+^ proliferating cells), Masson's trichrome staining (blue: collagen), and immunofluorescence (red: TUNEL^+^ apoptotic cells). Scale bar: 100 µm. Data are expressed as mean ± SD (n = 7). Statistical significance was assessed by two‐way ANOVA followed by Tukey's post hoc (B and D) or log‐rank (Mantel−Cox) test (E): ^****^ *p* < 0.0001; and ns, not significant.

Crucially, iGEL administration significantly extended skin allograft survival duration compared to CENP@GEL (CENP‐loaded hydrogel, 19.6 ± 1.1 days) (Figure S21). This result provides preliminary evidence for the combined effect between carvacrol prodrug‐mediated immunosuppression and SW033291‐induced regenerative enhancement. Compared to rapamycin, the standard‐of‐care immunosuppressive therapy, iGEL was superior to suppress acute skin allograft rejection under equivalent treatment frequency conditions, as evidenced by higher SGSSs and prolonged skin allograft survival duration (Figure S22). To further dissect the interactions of carvacrol and SW033291 as well as immune‐regenerative crosstalk, the efficacy of monotherapy was also evaluated in a murine skin transplantation model. The FD combination significantly prolonged skin allograft survival compared to single agents, while iGEL further enhancd this effect due to the optimized drug delivery performance (Figure S23A–C). This was accompanied by reduced levels of pro‐inflammatory cytokines (e.g., IL‐6, IL‐1β) and elevated levels of anti‐inflammatory cytokines (e.g., IL‐4, IL‐10) in skin allografts treated by iGEL (Figure S23D, E). Histopathological analysis revealed that carvacrol reduced lymphocyte infiltration in skin allografts, while SW033291 primarily promoted angiogenesis (Figure 4F and Figure S23F–I). The combination therapy, including FD and iGEL, showed reduced immune cell infiltration and enhanced vascular regeneration, suggesting that carvacrol‐mediated T‐cell suppression may alleviate immune interference during SW033291‐induced angiogenesis, while SW033291‐mediated tissue repair creates a favorable microenvironment for carvacrol's immunosuppressive function. To elucidate the regulatory mechanisms, qPCR analysis was performed on skin allografts treated with single or dual drug therapy. As expected, SW033291 dominated the regenerative gene expression changes (Figure S23J), while carvacrol primarily participated in immune regulation (Figure S23K). Specifically, combination therapy, especially iGEL, further enhanced this effect due to its improved drug delivery performance (Figure S23J,K).

### 3.4 iGEL Suppresses Alloreactive T‐Cell Responses and Modulates DC/Treg‐Associated Immune Regulation

Building on the efficacy of iGEL in improving skin allograft survival, we conducted histopathological and multiorgan flow cytometry analyses to investigate the immune mechanisms underlying its therapeutic effects, with particular focus on alloreactive T‐cell responses. On POD8, immunofluorescence staining revealed significant immune rejection in allogeneic skin grafts, characterized by marked infiltration of CD8^+^ and CD4^+^ T lymphocytes (Figure 5A and Figure S24). Importantly, iGEL treatment mitigated local immune responses more effectively than either FD or CSNP formulations, as evidenced by a significant reduction in T‐cell infiltration (Figure 5A). Peripheral immune organs (PIOs), particularly lymph nodes and the spleen, serve as the primary sites for T‐cell priming against donor alloantigens, driving the adaptive immune response during transplant rejection [20, 29]. Encouragingly, flow cytometry results showed a reduction in T‐cell proportions across gdLNs (Figure 5B), peripheral blood (Figure 5C), and the spleen (Figure S25), alongside a decrease in effector T‐cell populations and an increase in naive T‐cell frequencies (Figure 5D,E and Figure S26). Furthermore, iGEL treatment significantly increased circulating immunosuppressive regulatory T cells (Tregs) [20, 23], thereby protecting skin allografts from immune rejection (Figure 5F). Within PIOs and the bloodstream, dendritic cells (DCs) are critical for capturing and presenting alloantigens to naive T cells to initiate alloreactive immunity [30]. iGEL treatment significantly downregulated DC populations in both compartments (Figure 5G,H), suggesting potential modulation of antigen‐presentation pathways. The influence of iGEL on DC maturation was found to be quite limited, with only a downregulation of CD86 expression observed in the gdLNs within the GIM, suggesting a suppressed maturation of DCs (Figure S27). Besides, iGEL or CSNP treatment exerted minimal effects on natural killer (NK) cells, macrophage polarization, and neutrophil populations (Figure S28). In PIOs, including the spleen and gdLNs, iGEL had minimal impact on both M1 markers (CD80 and CD86) and the M2 marker (CD206) (Figure S29A–F). In peripheral blood, iGEL treatment showed a trend toward reduced CD86 expression and increased CD206 expression. However, these changes did not reach statistical significance compared with the saline group (Figure S29G–I). Together with the limited effects observed in lymphoid organs, these findings suggest that macrophage polarization is not a major contributor to the immunomodulatory activity of iGEL in the current model.

**FIGURE 5:**
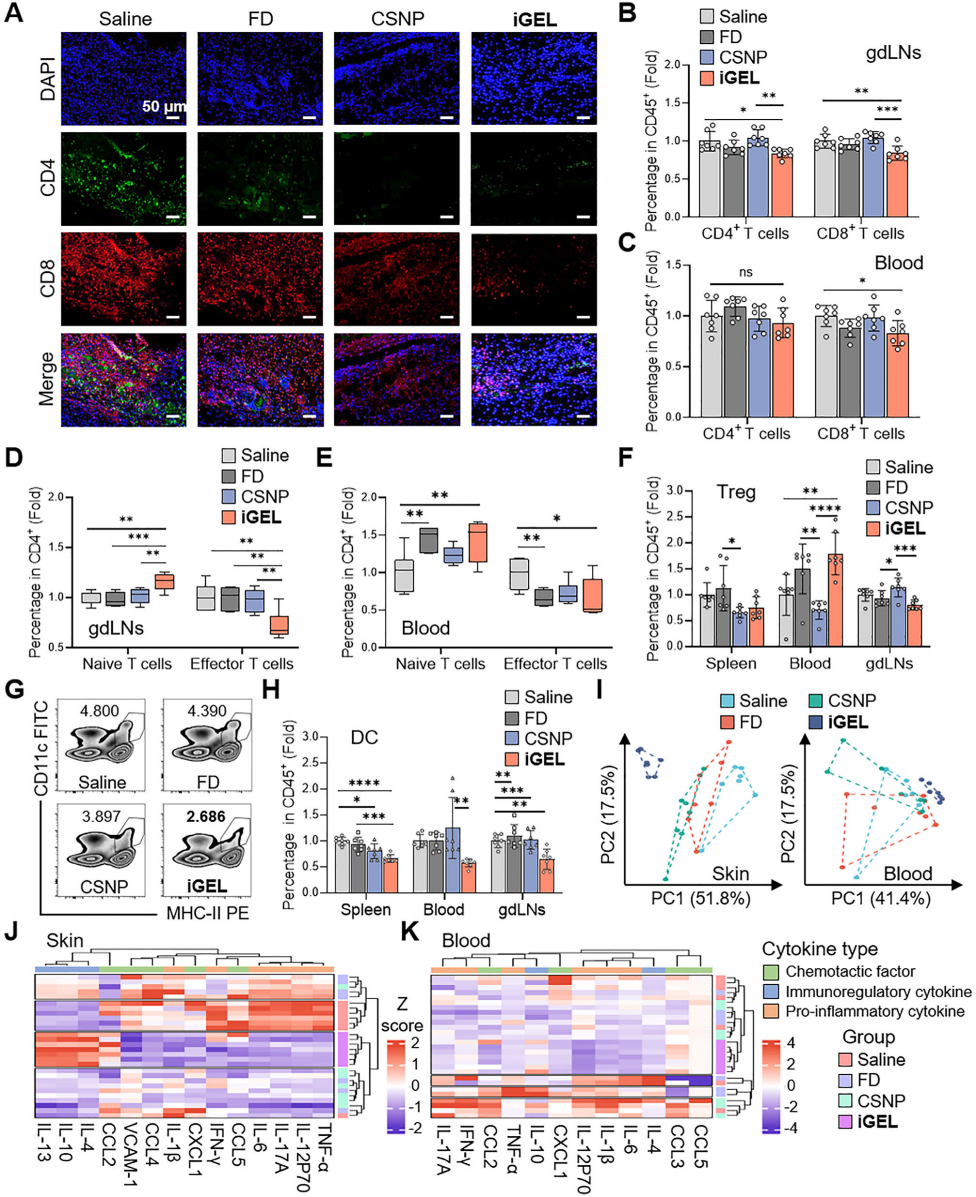
iGEL suppresses immune activation and remodels the skin allograft immune microenvironment. (A) Representative immunofluorescence images of skin allografts on POD8 (green: CD4^+^ T cells; red: CD8^+^ T cells). (B, C) Flow cytometric analysis of CD4^+^ and CD8^+^ T‐cell subsets within CD45^+^ populations from graft‐draining lymph nodes (gdLNs) and peripheral blood. (D, E) Frequencies of naive T cells (CD44^−^CD62L^+^) and effector T cells (CD44^+^CD62L^−^) within CD4^+^ populations from gdLNs and peripheral blood. (F) Regulatory T‐cell (CD4^+^Foxp3^+^) frequencies within CD45^+^ populations. (G, H) Quantification of dendritic cells (CD11c^+^MHC‐II^+^) in CD45^+^ populations, with representative flow cytometry plots. (I) PCA of cytokine profiles from skin allografts (14 cytokines) and peripheral blood (12 cytokines). (J, K) Hierarchical clustering heatmaps of cytokine expression in skin allografts and peripheral blood. Data are expressed as mean ± SD (n = 7). Statistical significance was assessed by one‐way ANOVA with Tukey's post–hoc test (B to F and H): ^*^ *p* < 0.05; ^**^ *p* < 0.01; ^***^ *p* < 0.001; ^****^ *p* < 0.0001 and ns, not significant.

Quantitative analysis of cytokines, key signaling molecules that regulate immune activation and inflammation, is essential for assessing the post‐transplant immune response [31]. To explore the cytokine landscape following iGEL implantation, we measured chemotactic factors, proinflammatory cytokines, and immunoregulatory molecules in both skin allografts and peripheral blood. PCA revealed distinct separation of cytokine profiles in the iGEL‐treated group compared to those in the other control groups (Figure 5I). Hierarchical clustering of Z‐score‐normalized cytokines revealed iGEL‐triggered changes in the intragraft cytokine patterns, marked by suppression of inflammatory mediators (e.g., IL‐6, IL‐1β) and simultaneous induction of immunoregulatory cytokines (e.g., IL‐4, IL‐10, IL‐13; Figure 5J). This shift suggests that iGEL promotes a Th2/Treg‐dominant phenotype in the GIM, which is known to suppress alloreactive T‐cell activation and encourage tolerance [32]. Despite its potent local immunomodulatory effects, iGEL showed a more limited impact on systemic immunity (Figure 5I). Systemic analysis of peripheral blood showed similar cytokine patterns in conventional treatment groups, while iGEL‐treated mice  maintained a distinct immunoregulatory profile. This signature included systemic suppression of IL‐1β, TNF‐α, and IL‐12P|70 (Figure 5K), which are known to be crucial in driving DC maturation and Th1 polarization—processes that enhance alloimmune responses [33, 34]. In conclusion, these findings suggest that iGEL modulates both local and systemic cytokine balances, thereby creating a skin allograft‐protective immune environment.

### 3.5 iGEL Facilitates Tissue Regeneration for Improved Transplant Longevity

To investigate the mechanisms driving iGEL‐enhanced skin allograft survival, we performed transcriptomic profiling of skin allografts following treatment. By applying thresholds of |fold change| > 2 and *p* < 0.05, we identified 600 differentially expressed genes (DEGs) between the iGEL and saline groups and 337 DEGs between the iGEL and CSNP treatments (Figure 6A,B and Figure S30). GO enrichment analysis revealed that these DEGs were mainly linked to processes involved in epidermal cell and keratinocyte differentiation (Figure 6C,D), suggesting favorable restoration of barrier function, which is crucial for skin allograft viability. In addition to epidermal remodeling, iGEL triggered significant reprogramming of the dermal microenvironment. Cellular component analysis revealed an enrichment of collagen‐containing extracellular matrix (Figure S31), indicating active dermal matrix synthesis. Furthermore, molecular functions related to enzyme inhibitor activity (modulating ECM degradation), glycosaminoglycan/heparin binding (facilitating growth factor sequestration), and sulfur compound binding (possibly regulating redox balance) were notably enriched (Figure S31). These results suggest that iGEL acts as a regulator of tissue homeostasis, promoting both epidermal barrier repair and dermal matrix stabilization to establish a regenerative niche that mitigates alloimmune attack.

**FIGURE 6:**
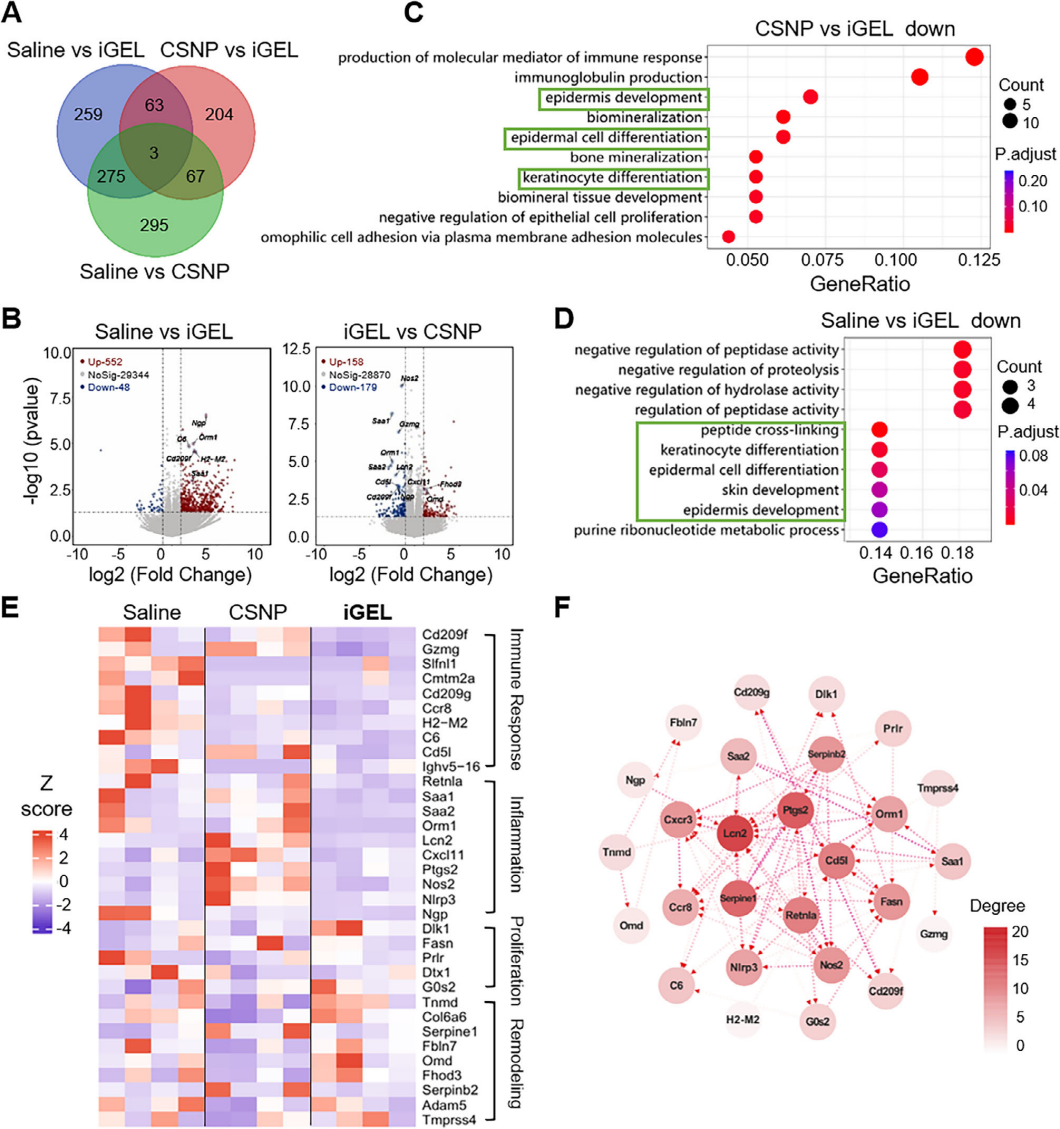
Transcriptome sequencing of allogeneic skin transplantation models. (A) Venn diagram of shared and unique genes across three experimental groups. (B) Volcano plot of differentially expressed genes (DEGs) between groups (blue: downregulated; red: upregulated; gray: unchanged). (C and D) GO enrichment analysis of DEGs. (E) Heatmap of DEGs in skin allografts (|FoldChange| > 2, *p* < 0.05). (F) Cytoscape‐based network analysis of DEGs.

Based on the dynamic biological processes involved in skin allograft integration with the host, DEGs—especially those overlapping across multiple comparisons—were categorized into four functional phases: immune response, inflammation, proliferation, and remodeling. Compared to saline or CSNP controls, iGEL treatment significantly reduced the expression of immune‐inflammatory genes (Figure 6E). Cytoscape‐based network analysis further revealed strong interconnectivity among the DEGs (Figure 6F). This coordinated downregulation within a tightly interconnected network strongly supports the role of iGEL in establishing a local immunosuppressive GIM. In skin allograft, the integration of proliferation and remodeling is essential for restoring structural and functional integrity to ensure long‐term skin allograft viability. Notably, iGEL enhanced the expression of genes such as Delta‐like 1 homolog (Dlk1, which promotes re‐epithelialization and angiogenesis [35]) and Tenomodulin (*Tnmd*, which supports collagen synthesis [36]) within this phase, indicating a therapeutically induced program of functional tissue remodeling. Together, these findings highlight the effectiveness of locoregional activation of the iGEL binary delivery system in promoting skin allograft integration.

### 3.6 iGEL Enhances Neovascularization and Collagen Restructuring to Accelerate Acute Wound Healing

To directly validate the activity of iGEL in tissue repair, we established a murine full‐thickness skin wound model (Figure 7A) and compared wound healing progression following treatments with carvacrol or SW033291 monotherapy, dual FD combination, CSNP, and iGEL (Figure 7B,C). Longitudinal gross observation and quantitative analysis revealed similar wound closure rates across all groups at POD3 (Figure S32). While the CSNP group showed significantly accelerated healing with closure rates of 63.5% ± 3.0% and 81.0% ± 3.9% at POD6 and 9, respectively, neither monotherapy nor combination therapy demonstrated statistically enhanced efficacy compared to the saline controls (42.8% ± 13.1% at POD6 and 61.1% ± 3.3% at POD9) (Figure 7D,E). Notably, iGEL treatment achieved superior regenerative performance, with 88.1% ± 2.6% wound closure by POD9, outperforming both saline controls and the CSNP group, thereby highlighting its substantial drug delivery capacity. To further assess healing progression, wound tissues harvested at POD9 were subjected to histopathological evaluation. Re‐epithelialization is crucial for restoring the functional barrier in full‐thickness skin wounds through epidermal continuity. Consistently, both CSNP and iGEL treatments significantly enhanced re‐epithelialization compared to the saline controls, with iGEL‐treated wounds exhibiting maximal epithelial thickness, robust cell proliferation, and the highest neovascular density (Figure 7F,G). Collagen deposition is key to restoring wound tensile strength by providing structural scaffolding during tissue remodeling. Quantitative analysis revealed markedly higher collagen deposition in the iGEL group, considerably aiding skin restoration (Figure 7H). In conclusion, these results demonstrate the regenerative capacity of iGEL to enhance angiogenesis and extracellular matrix remodeling, accelerating full‐thickness wound healing beyond conventional delivery systems.

**FIGURE 7:**
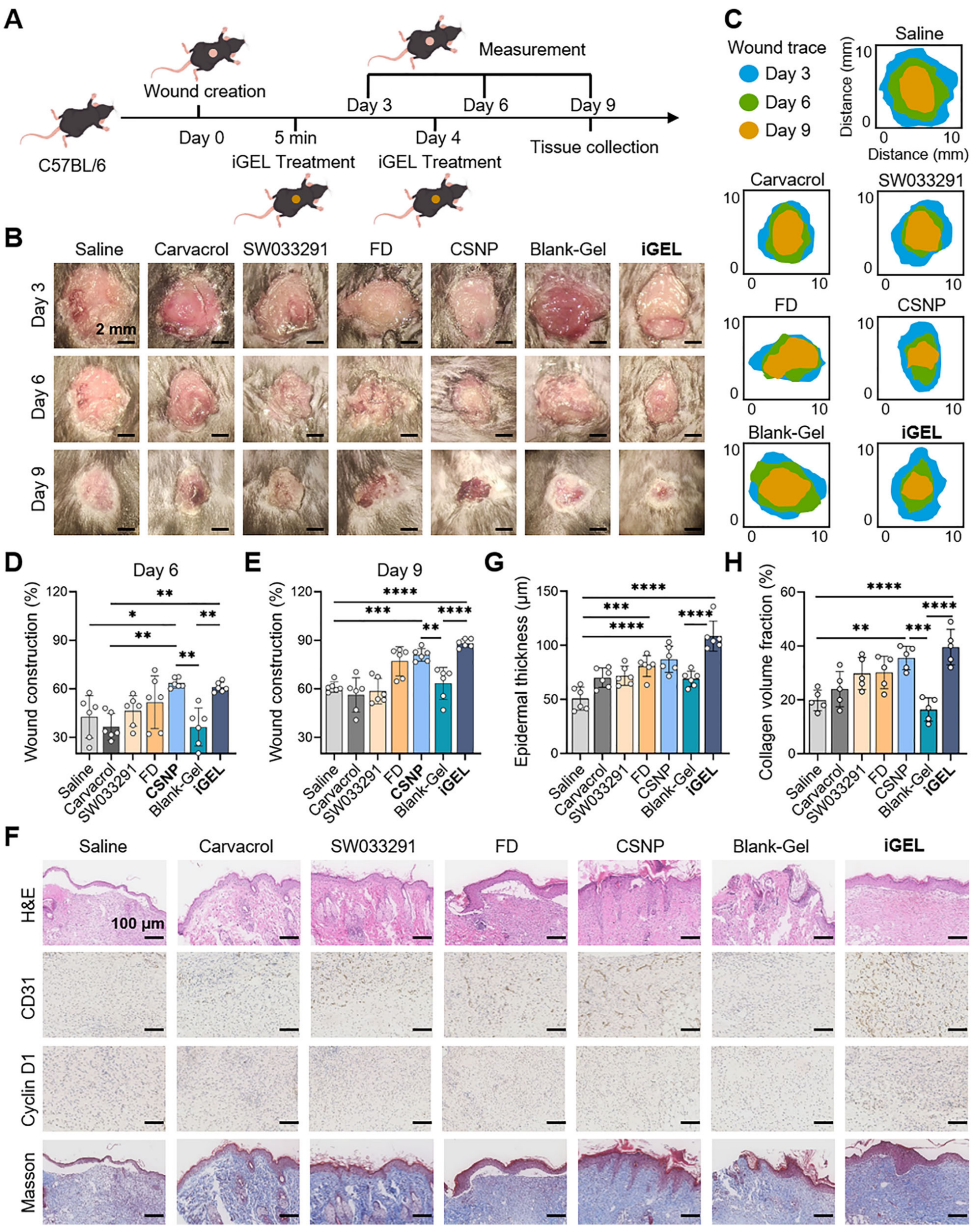
Therapeutic efficacy of iGEL in acute wound healing. (A) Schematic of the experimental design. (B) Representative wound images from each group at POD3, POD6, and POD9. Scale bar: 2 mm. (C) Progression of wound closure. (D, E) Quantitative analysis of wound healing rates on days 6 and 9. (F) Histological assessment of wound tissues at POD9: H&E, CD31/Cyclin D1 IHC, and Masson's trichrome staining. (G, H) Quantification of re‐epithelialization thickness and collagen deposition at POD9. Scale bar: 100 µm. Data are expressed as mean ± SD (n = 6 in D, E, and G; n = 5 in H). Statistical analysis was performed by one‐way ANOVA with Tukey's test (D, E, G, and H): ^*^ *p* < 0.05; ^**^ *p* < 0.01; ^***^ *p* < 0.001; and ^****^ *p* < 0.0001.

### 3.7 iGEL Mitigates Graft Rejection and Facilitates Tissue Remodeling in Diabetic Transplant Mice

Based on the promising therapeutic effects observed in allogeneic skin grafts and incisional wound healing models with immunocompetent, metabolically stable recipients, we expanded our research to include clinically relevant diabetic transplant recipients. Diabetic hosts create a particularly challenging environment for skin allograft integration, marked by vascular dysfunction, Th1/Th2 imbalance, Treg cell dysfunction, and reduced regenerative capacity [37, 38, 39]—factors that together significantly hinder transplant survival. Diabetes was induced through consecutive streptozotocin injections, followed by allogeneic skin transplantation and iGEL implantation once stable diabetic hyperglycemia was confirmed (Figure 8A,B). By POD8, all groups, except the iGEL group, showed typical rejection signs, such as considerable shrinkage, crusting, and sclerosis (Figure 8C). Quantitative analysis of SGSSs, based on necrotic areas, indicated significantly higher scores in the iGEL group compared to those of the other treatment groups (Figure 8D), with nearly double the median skin allograft survival (17.1 ± 2.2 days) compared to saline controls (9.1 ± 0.7 days; Figure 8E). Treatment with FD (11.9 ± 1.5 days) or CSNP (13.0 ± 1.7 days) moderately prolonged skin allograft survival. Notably, iGEL implantation exerted no significant effect on body weight despite progressive diabetes‐associated weight loss (Figure 8F). Histological analysis revealed that iGEL treatment preserved superior skin allograft architecture, with well‐organized epidermal and dermal layers and minimal lymphocytic infiltration. Alternatively, saline controls exhibited severe structural disruption and dense immune cell aggregation (Figure 8G). Furthermore, iGEL‐treated skin allografts demonstrated significantly increased CD31‐positive microvessel density and well‐organized collagen fiber deposition (Figure 8G and Figure S33). These findings demonstrate that iGEL exerts effective immune modulation and tissue repair capabilities, even within the challenging diabetic microenvironment, which is complicated by impaired blood perfusion and elevated immune responses.

**FIGURE 8:**
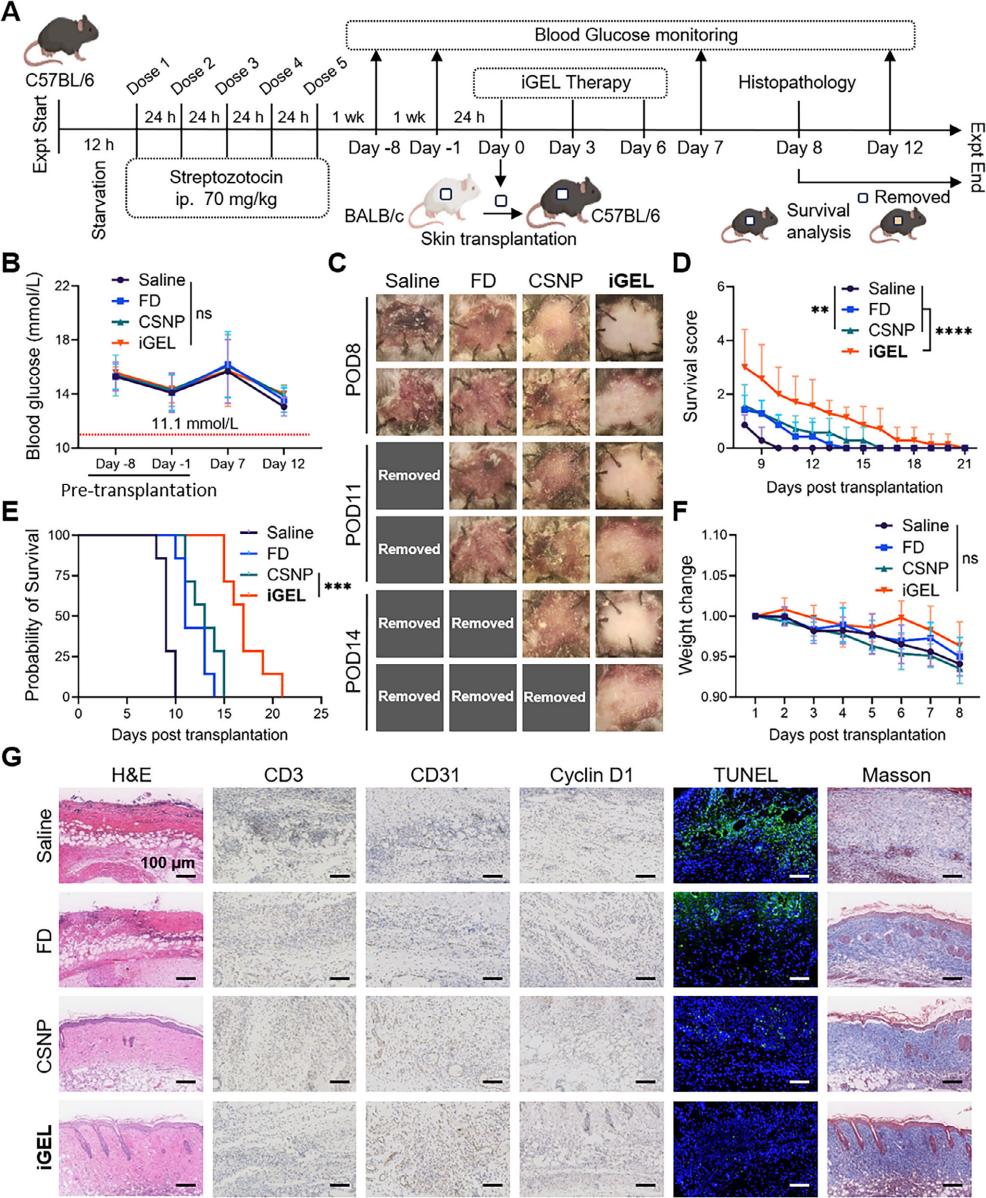
Functional evaluation of iGEL in diabetic mouse skin transplantation. (A) Schematic of the experimental workflow. (B) Longitudinal monitoring of blood glucose levels. (C) Representative macroscopic morphology of skin allografts in different groups. (D) Quantitative skin allograft survival scores from POD8 to rejection. (E) Kaplan–Meier survival analysis of skin allografts. (F) Temporal body weight changes during treatment. (G) Histopathological analysis at POD8 (green: TUNEL^+^ apoptotic cells). Scale bar: 100 µm. Data are expressed as mean ± SD (n = 7). Statistical significance was assessed by two‐way ANOVA followed by Tukey's post hoc (B, D, and F) or log‐rank (Mantel−Cox) test (E): ^**^ *p* < 0.01; ^***^ *p* < 0.001; ^****^ *p* < 0.0001; and ns, not significant.

### 3.8 Safety Assessment in Vivo

For a clearer understanding of the clinical translation potential of iGEL, a thorough biosafety evaluation was conducted on healthy C57BL/6 mice (Figure S34A). This evaluation included a multiparameter analysis of acute toxicity, histocompatibility at implantation sites, and systemic immunogenicity responses. The mice received carvacrol (30 or 45 mg/kg dose‐equivalent) and SW033291 (10 or 15 mg/kg dose‐equivalent) through different routes. No significant body weight loss was observed in any treatment group (Figure S34B). Biochemical analyses of alanine aminotransferase (ALT), aspartate aminotransferase (AST), creatinine (CR), and blood urea nitrogen (BUN) post‐treatment showed no evident hepatorenal toxicity, even at therapeutic or elevated doses (Figure S34C–F). Hematological tests confirmed no adverse effects on RBCs, white blood cells (WBCs), platelet (PLT) counts, or hemoglobin (HGB) levels (Figure S34G–J). Histopathological examinations of the heart, liver, spleen, lung, and kidney tissues revealed no pathological changes, such as cellular necrosis, inflammatory infiltration, or fibrosis (Figure S34K). To assess long‐term safety, healthy C57BL/6 mice were evaluated at post‐treatment 30 and 50 days, showing no hepatic or renal dysfunction (Figure S35A–D). Importantly, no significant inflammatory infiltrates or tissue damage were observed throughout the implantation period (Figure S35E), collectively supporting the hydrogel's safety profile and feasibility for prolonged application.

To further evaluate the safety of iGEL under transplantation‐associated inflammatory conditions, we performed additional assessments in an allogeneic skin transplantation model. Liver and kidney function tests were conducted at post‐transplantation day 10 (during active allograft presence) and day 30 (after allograft loss). No significant abnormalities in ALT, AST, BUN, or CR were observed at either time point compared with either saline‐treated transplanted mice or age‐matched healthy controls (Figure S36A–H). Body weight remained stable across all groups (Figure S36I). These results demonstrate that iGEL maintains a favorable safety profile even under inflammatory transplantation conditions. Overall, these thorough analyses affirm the biosafety of iGEL for potential clinical use.

## 4 Discussion

While graft acceptance rates have improved significantly because of recent advancements in transplant surgery [15], long‐term survival still remains suboptimal. This is largely due to the limitations of conventional systemic immunosuppression, which includes severe off‐target toxicity and inadequate drug accumulation at the graft site [8, 40]. Traditional therapies often fail to deliver site‐specific immunomodulation, leading to poor tissue repair and increased risks of infection or metabolic complications [13, 41, 42]. To overcome these challenges, new hydrogel‐based drug delivery systems have been developed. These systems offer sustained drug retention at target sites [42, 43, 44], support various therapeutic agents with stimuli‐responsive release mechanisms, and, owing to their moldable properties, provide conformal coverage of irregular surgical cavities, addressing the limitations of rigid implants.

Herein, we introduced a novel dual‐targeting strategy to simultaneously address immune rejection and tissue regeneration—marking a paradigm shift beyond traditional immunosuppression‐focused approaches [5, 45]. Clinically, the failure to address post‐transplant regeneration has contributed to suboptimal outcomes, leaving grafts vulnerable to small‐for‐size syndrome and fibrosis due to poor vascularization and maladaptive remodeling [10, 46, 47]. iGEL overcame these challenges through the coordinated integration of immunomodulatory and regenerative therapies enabled by modular nanotherapy and microenvironment‐responsive drug delivery. UFA‐functionalized prodrugs improve drug stability and solubility [48, 49], forming self‐assembled pharmacological nanoparticles that coencapsulate immunosuppressive and regenerative agents into a modular nanodrug reservoir. These nanodrugs were embedded within an inflammation‐responsive hydrogel scaffold using dynamic TSPBA‐PVA boronic ester bonds, enabling initiator‐free gelation and spatiotemporally controlled release. The hydrogels composed of TSPBA/PVA offer the advantages of pathological responsiveness and have been extensively studied for therapeutic delivery such as drugs [22], mRNA [50], and cells [51], with tunable mechanical properties achievable through concentration adjustments. The hydrogel's in situ delivery maintains therapeutic efficacy, reduces dosing frequency, and shows a favorable biocompatibility profile in both healthy and transplantation‐associated inflammatory settings, while reducing systemic exposure and potential side effects. This integrated design transformed the GIM from a hostile state to a regenerative one by suppressing harmful immune responses and promoting functional tissue reconstruction.

The unique immunological characteristics of skin as a barrier organ, characterized by coordinated immune responses involving epidermal, dermal, and subcutaneous cells [52]. The inherent immunological challenges of allogeneic skin transplantation, characterized by robust innate and adaptive immune activation leading to graft destruction, inherently limit long‐term survival [53, 54]. Indeed, skin transplantation is widely regarded as one of the most stringent models in transplantation immunology because of its high density of resident antigen‐presenting cells and continuous exposure to environmental stimuli. Consequently, even relatively modest extensions in graft survival may represent biologically meaningful alterations in the local immune‐regenerative microenvironment and provide a stringent benchmark for evaluating emerging transplant therapeutics. For instance, immunoprotective vesicle‐crosslinked hydrogels achieved only 60% survival at 14 days [20], while our prior micelle [55] or self‐assembled nanoparticle [56] approaches extended survival to ∼30 days only with prolonged dosing. In contrast, iGEL effectively mitigated immune rejection by suppressing effector T cells and restoring inflammatory balance, thus prolonging skin allograft survival with just three treatments. In addition to inhibition of T cells, carvacrol is also reported to hinder M1‐type macrophages polarization by activating Wnt signaling [23]. Although the short‐term, intermittent administration of iGEL demonstrated limited effects on macrophage polarization, the dose‐ and duration‐dependent relationships of carvacrol in modulating this process require further investigation. Besides, chronic rejection is characterized by humoral immune responses mediated by donor‐specific antibody activity [57, 58], for which iGEL exhibits limited efficacy. Prioritizing the screening of B cell‐regulatory molecules and optimizing synthesis processes to develop a sustained‐release platform tailored for managing chronic rejection represents a promising research direction.

In addition to exerting a strong suppressive effect on acute rejection responses, iGEL promoted tissue regeneration by enhancing vascularization and collagen remodeling, supporting the reconstruction of functional skin allograft architecture [59, 60]. The proregenerative potential of iGEL was further confirmed in acute wound models, where it accelerated re‐epithelialization, demonstrating its dual therapeutic potential. Notably, iGEL alone achieved sustained skin allograft survival and functional repair while minimizing the need for systemic immunosuppression, presenting a promising strategy to balance therapeutic efficacy with long‐term safety in transplantation. Like other hydrogel‐based modalities [20, 22, 61] designed to minimize systemic exposure, complete prevention of systemic dissemination of locally administered drugs remains technically unachievable at present. As expectation, local iGEL implantation also led to detectable systemic immune modulation, which likely arises from trace drug leakage via lymphatic or circulatory pathways. Although a minor degree of systemic distribution following local implantation cannot be completely excluded, biodistribution analyses demonstrated preferential accumulation of therapeutics within skin allografts and gdLNs. Therefore, the collective pharmacokinetic, biodistribution, and mechanistic findings support localized inflammation‐responsive drug delivery as the predominant driver of therapeutic efficacy, although a minor contribution from residual systemic exposure cannot be completely excluded.

Additionally, we extended our evaluation of iGEL to a clinically relevant diabetic skin transplantation model, where hyperglycemia and impaired vascularization worsen immune rejection and reduce skin allograft survival [38, 39]. In these metabolically dysregulated conditions, iGEL nearly doubled the median skin allograft survival while restoring functional vascular networks and reorganizing collagen architecture. These findings highlight unique ability of iGEL to both counteract immune‐mediated destruction and reverse tissue maladaptation, even under the combined stresses of hyperglycemia and ischemia. Unlike conventional immunosuppressive hydrogels or barrier‐based systems [20, 62], iGEL utilizes inflammation‐triggered drug release to dynamically synchronize therapeutic action with disease progression. This provides a pathology‐responsive solution that silences destructive immunity while promoting functional tissue restoration. This approach not only advances transplant therapeutics but also sets the stage for innovative biomaterial designs in complex inflammatory conditions, where dual targeting of immune and stromal compartments is essential for functional recovery. However, further refinements will likely be required when extending iGEL to vascularized solid‐organ transplantation. Future optimization may focus on enhancing tissue adhesion, mechanical stability, and local retention within highly perfused environments while preserving inflammation‐responsive degradation and controlled drug release. In addition, modulation of hydrogel porosity and degradation kinetics may facilitate more uniform therapeutic distribution throughout larger and structurally complex graft tissues. From a translational perspective, image‐guided peri‐graft administration and organ‐conformal implantation strategies may further improve spatial localization and therapeutic coverage in liver and kidney transplantation, facilitating the extension of this proof‐of‐concept platform to clinically relevant solid‐organ transplant settings.

The iGEL platform showed strong efficacy in reducing skin allograft rejection and promoting tissue repair in murine skin transplantation and acute wound healing models. However, several challenges must be addressed to enhance its clinical applicability. First, although iGEL showed a favorable safety profile in healthy mice and in the allogeneic skin transplantation model, its long‐term safety in vascularized solid‐organ transplantation remains to be systematically evaluated. In particular, potential risks related to chronic inflammation, fibrotic encapsulation, and organ‐specific tissue responses should be further investigated before clinical translation. Second, the current validation focused on superficial skin allografts; hence, the efficacy of the platform in deep or irregularly shaped transplants remains untested. In such cases, mechanical stress and uneven ROS distribution may influence drug release kinetics. Third, although inflammation‐triggered release facilitates microenvironment‐responsive delivery, the absence of a clear on/off switching mechanism could limit precision in fast‐evolving clinical situations. Fourth, the pathological heterogeneity of GIM across different transplanted organs, individual patients, and even intra‐organ regions results in marked variability in key parameters (e.g., pH, ROS profiles, enzymatic signatures), rendering single‐stimulus‐responsive hydrogels clinically insufficient for achieving consistent high response rates. Fifth, Chronic implantation further risks foreign body reactions leading to fibrous capsule formation, with implant geometry and dimensions critically modulating fibrotic severity, as demonstrated by Veiseh et al. who showed that optimizing implant size and shape significantly reduces such responses [63]. Finally, engineering synthetic materials to confer “smart” responsiveness to pathological cues (e.g., pH, ROS, enzymes) often results in potential excipient‐associated toxicity risks and may compromise further clinical translation [64]. Future iterations could incorporate multistimuli‐responsive chemistries to improve spatiotemporal control, ensuring adaptability to complex graft microenvironments.

In conclusion, we developed an inflammation‐responsive hydrogel embedded with chemically engineered lipidic prodrug nanoassemblies, which integrates locoregional immunosuppression and regenerative repair within a single therapeutic platform to advance transplant therapy. This injectable platform enables controlled drug delivery to the GIM, providing a dual‐action approach that outperforms conventional systemic therapies by combining localized immunomodulation with regenerative support. By utilizing inflammation‐triggered drug activation, iGEL minimizes off‐target toxicity and adapts to various graft anatomies, offering a clinically translatable strategy for improving allograft outcomes, particularly in highly immunogenic transplantation settings. Additionally, its sprayable design and microenvironment‐targeted reprogramming present a clinically flexible strategy to improve allograft survival, merging precision immunology with regenerative engineering for better patient outcomes.

## Author Contributions

Conceptualization: X.H.Y., W.H.X., and Z.S.S. Methodology: W.N. and S.R.Q. Investigation: F.Y., W.Z.H., and T.X.Y. Resources: X.H.Y. and S.P.H. Funding acquisition: X.H.Y., Z.S.S., and Z.K. Data curation: T.H., Z.W.T., and L.Z. Validation: Z.J.T., and G.Y.N. Formal analysis: Z.J.T. Supervision: X.H.Y. Project administration: X.H.Y. and Z.S.S. Visualization: W.N. and S.R.Q. Writing – original draft: W.N. Revision of the manuscript: W.N. and S.R.Q.

## Funding

This work was supported by “Pioneer” and “Leading Goose” R&D Program of Zhejiang (No. 2025C04007), National Natural Science Foundation of China (No. 32171368, 82302004, and 82160128), Non‐profit Central Research Institute Fund of Chinese Academy of Medical Sciences (No. 2023‐PT320‐02). Zhejiang Provincial Natural Science Foundation of China (LQ24H100003).

## Conflicts of Interest

The authors declare no conflicts of interest.

## Supporting information




**Supporting File**: advs76422‐sup‐0001‐SuppMat.docx.

## Data Availability

All data needed to evaluate the conclusions in the paper are present in the paper and/or the Supplementary Materials.
