# Supplementary material for: Engineering Immunoregenerative Therapy via an Immunomodulatory Binary Pharmacology Hydrogel Depot for Prolonged Allograft Survival
Source: Adv Sci (Weinh). 2026 Jul 7:e20994. Online ahead of print. doi: 10.1002/advs.202520994 (PMC13339004; doi:10.1002/advs.202520994)
Supplement: Supplementary file 1 — Supporting File: advs76422‐sup‐0001‐SuppMat.docx. [file ADVS-9999-e20994-s001.docx]

**Supporting Information**

***Engineering immunoregenerative therapy via an immunomodulatory binary pharmacology hydrogel depot for prolonged allograft survival***

Ning Wang^1, 2, †^, Ruiqi Sun^1, 2, †^, Yang Fu^1, 2^, Zhonghan Wu^3^, Xinyu Tong^1, 2^, Hong Tang^1, 2^, Wentao Zhao^1, 2^, Zhi Liang^1, 2^, Jintao Zheng^1, 2^, Yanan Guan^1, 2^, Ke Zhou^3^, Penghong Song^1, 2^, Shusen Zheng*****^1, 2^, Hangxiang Wang*****^1, 2^, Haiyang Xie*****^1, 2^

1Division of Hepatobiliary and Pancreatic Surgery, Department of Surgery, The First Affiliated Hospital, Zhejiang University School of Medicine, Hangzhou 310003, China

2NHC Key Laboratory of Combined Multi-organ Transplantation, Key Laboratory of Organ Transplantation, Zhejiang Province. Institute of Organ Transplantation, Zhejiang University. State Key Laboratory for Diagnosis and Treatment of Infectious Diseases, National Clinical Research Center for Infectious Diseases, Hangzhou 310003, China

3Division of Lung Transplantation and Thoracic Surgery, Department of Surgery, The First Affiliated Hospital, Zhejiang University School of Medicine, Hangzhou 310003, China

†These authors contributed equally to this work.

*Corresponding author. E-mail: xiehy@zju.edu.cn, E-mail: wanghx@zju.edu.cn, Email: shusenzheng@zju.edu.cn.

**Evaluation of the Immunosuppressive Interactions Between Carvacrol and SW033291 in Activated T Cells**

Primary T lymphocytes were isolated from the spleens of C57BL/6 mice using a Mouse T Cell Isolation Kit according to the manufacturer's instructions. Purified T cells were cultured in complete RPMI-1640 medium supplemented with recombinant mouse IL-2 (2 ng/mL, RP01384, ABclonal), anti-mouse CD3 antibody (100340, BioLegend), and anti-mouse CD28 antibody (102116, BioLegend) for 48 h to induce T-cell activation. Activated T cells were subsequently treated with carvacrol (20, 30, or 50 μM) and SW033291 (5, 7.5, or 12.5 μM), either alone or in combination, for an additional 48 h. Following drug treatment, cells were stimulated with a Cell Activation Cocktail (423304, BioLegend) for 4 h to facilitate intracellular cytokine accumulation. Cells were then stained with fluorochrome-conjugated antibodies against CD45 (BV510, 563891, BD Biosciences), CD3e (BV650, 564378, BD Biosciences), CD4 (PE-Cy7, 100422, BioLegend), and CD8a (Alexa Fluor 700, 557959, BD Biosciences) for surface marker analysis. After fixation and permeabilization using the Cyto-Fast™ Fix/Perm Buffer Kit (426803, BioLegend), intracellular staining was performed using antibodies against IFN-γ (APC, 505810, BioLegend), granzyme B (PE, 168804, BioLegend), and perforin (FITC, 154310, BioLegend). Samples were analyzed by flow cytometry, and the mean fluorescence intensity (MFI) of IFN-γ in CD4⁺ and CD8⁺ T cells was quantified. The inhibitory interactions between carvacrol and SW033291 were evaluated by calculating the Combination Index (CI) using Calcusyn software.

**Encapsulation Efficiency of CSNP**

Post-synthesis, CSNP nanoparticles underwent ultracentrifugation (Beckman XPN-100, 100,000 × g, 60 min) to separate free drug. The supernatant was then treated with 0.1 M NaOH (37°C, 4 h) to hydrolyze unencapsulated drug, neutralized with 0.1 M HCl, and quantified by High-performance liquid chromatography (HPLC) to calculate encapsulation efficiency.

**Evaluation of iGEL Release Kinetics in T lymphocyte-conditioned Media**

To explore the physiological relevance of drug release assays, a murine allogeneic skin transplantation model was established to validate the inflammatory microenvironment. On post-transplant day 8, gdLN lymphocytes were isolated and cultured for 4 days. T cell activation was further enhanced using Leukocyte Activation Cocktail (550583, BD Biosciences). For release kinetics assessment, 1 mL iGEL (3.75 mg carvacrol equivalents + 1.25 mg SW033291) was sealed in 7 kDa dialysis bags and immersed in T lymphocyte-conditioned media under 37°C shaking incubation. External media were sampled at scheduled intervals and replaced with fresh media. Samples were treated with 0.1 M NaOH (37°C, 4 h) and neutralized with 0.1 M HCl before HPLC analysis.

**Evaluation of physical and mechanical properties of iGEL**

To systematically evaluate the clinical applicability of the materials, injection force tests were conducted using a Zwick/Roell Z010 universal testing machine, with a constant injection rate of 1 mL/min via a clinically standard 1 mL sterile syringe equipped with a 27G needle. Three independent biological replicates were tested for each group at every time point (0 h, 2 h, 4 h). Rheological characterization included shear rate sweep tests to assess viscosity stability under clinically relevant shear rates (~3700 s⁻¹, corresponding to 1 mL/min injection through a 27G needle), three-step thixotropic cycle tests (1 s⁻¹ → 500 s⁻¹ → 1 s⁻¹ shear) to simulate injection and evaluate shear recovery, and dynamic time sweep tests (1 Hz, 1% strain within the linear viscoelastic range) to confirm the formation and stability of the iGEL hydrogel network. Negative viscosity values at extremely low shear rates (<1 s⁻¹) were attributed to instrumental background noise as the dilute solutions approached the torque detection limit. Swelling assays were performed using a wet-state in-situ method, where fully crosslinked iGEL discs were pre-equilibrated in pH 7.4 PBS at 37 °C to reach initial hydration equilibrium (recorded as initial wet weight). Subsequent weight changes were monitored under different pH conditions (7.4, 6.5, 5.0, and 8.0) to mimic physiological and pathological graft immune microenvironments (GIM) post-transplantation, as well as under 10 mM H₂O₂ to simulate high ROS levels during acute allograft rejection.

**Synthesis of carvacrol prodrugs**

1. Synthetic scheme of Car-EPA prodrug.
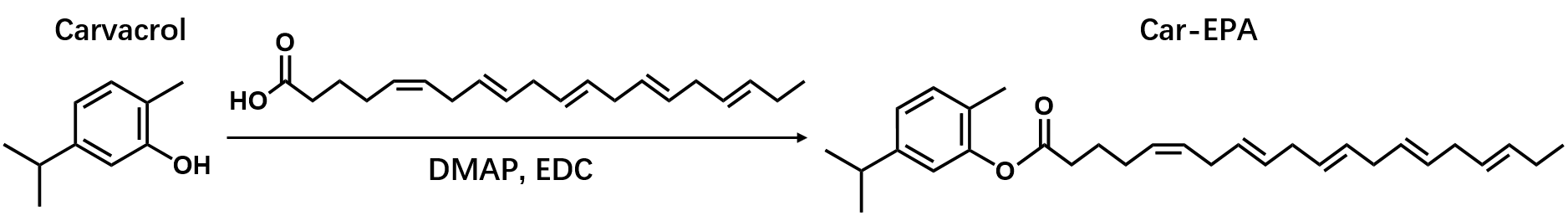


Carvacrol (1.0 eqv., 100.0 mg, 0.67 mmol), eicosapentaenoic acid (1.2 eqv., 241.6 mg, 0.8 mmol), DMAP (1.2 eqv., 122 mg, 0.8 mmol) and EDC (1.2 eqv., 155 mg, 0.8 mmol) were mixed and dissolved in about 2 mL ultra-dry dichloromethane (DCM). The reaction solution was stirred and heated at 45 ℃ overnight on the magnetic hotplate stirrer (Heidolph, Germany). The reaction progress was monitored using thin-layer chromatography (TLC). Upon completion of the reaction, the reaction mixture was cooled and subsequently washed with 5% w/w citric acid, saturated aqueous NaHCO_3_, and brine. The organic layer was dried over anhydrous sodium sulfate and then evaporated under rotary evaporation. The product was further subjected to purification using column chromatography on silica gel in DCM as the mobile phase to obtain Car-EPA (170.7 mg, 58.7%). The purified product was subsequently subjected to structural analysis using ^1^H nuclear magnetic resonance (NMR).

2. Synthetic scheme of Car-DHA prodrug.


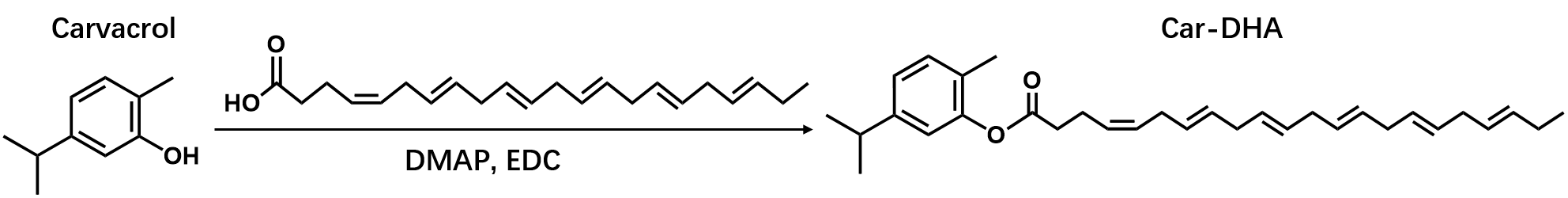


Carvacrol (1.0 eqv., 100.0 mg, 0.67 mmol), docosahexaenoic acid (1.2 eqv., 262.4 mg, 0.8 mmol), DMAP (1.2 eqv., 122 mg, 0.8 mmol) and EDC (1.2 eqv., 155 mg, 0.8 mmol) were mixed and dissolved in about 2 mL ultra-dry DCM. The reaction solution was stirred and heated at 45 ℃ overnight on the magnetic hotplate stirrer. The reaction progress was monitored using TLC. Upon completion of the reaction, the reaction mixture was cooled and subsequently washed with 5% w/w citric acid, saturated aqueous NaHCO_3_, and brine. The organic layer was dried over anhydrous sodium sulfate and then evaporated under rotary evaporation. The product was further subjected to purification using column chromatography on silica gel in DCM as the mobile phase to obtain Car-DHA (154.7 mg, 50.2%). The purified product was subsequently subjected to structural analysis using ^1^H NMR.

3. Synthetic scheme of Car-OA prodrug.


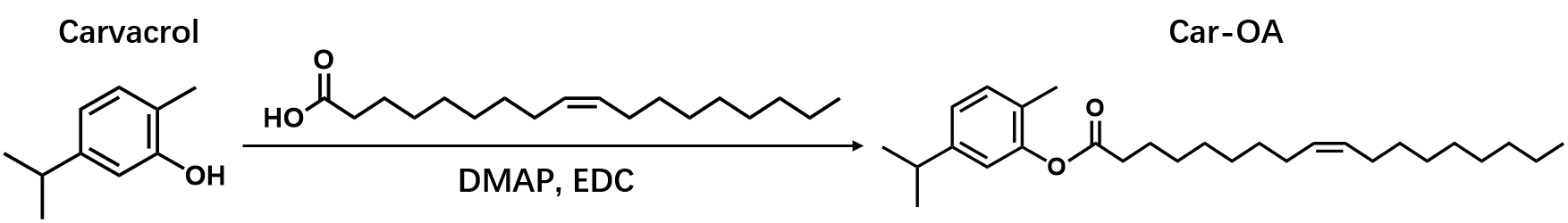


Carvacrol (1.0 eqv., 100.0 mg, 0.67 mmol), oleic acid (1.2 eqv., 225.6 mg, 0.8 mmol), DMAP (1.2 eqv., 122 mg, 0.8 mmol) and EDC (1.2 eqv., 155 mg, 0.8 mmol) were mixed and dissolved in about 2 mL ultra-dry DCM. The reaction solution was stirred and heated at 45 ℃ overnight on the magnetic hotplate stirrer. The reaction progress was monitored using TLC. Upon completion of the reaction, the reaction mixture was cooled and subsequently washed with 5% w/w citric acid, saturated aqueous NaHCO_3_, and brine. The organic layer was dried over anhydrous sodium sulfate and then evaporated under rotary evaporation. The product was further subjected to purification using column chromatography on silica gel in DCM as the mobile phase to obtain Car-OA (145.1 mg, 52.3%). The purified product was subsequently subjected to structural analysis using ^1^H NMR.

4. Synthetic scheme of Car-LA prodrug.


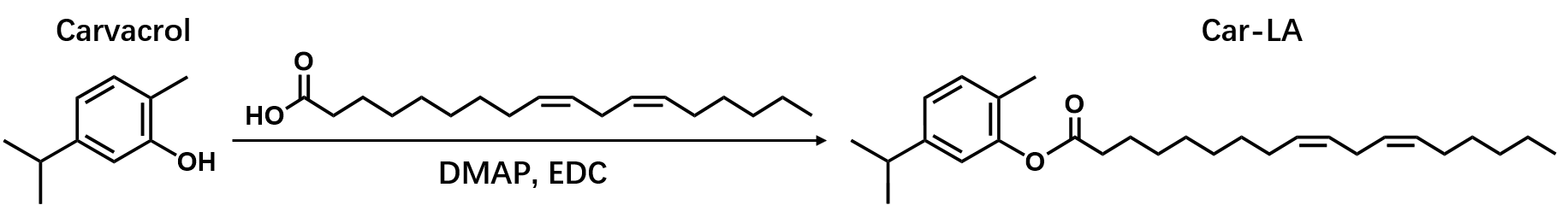


Carvacrol (1.0 eqv., 100.0 mg, 0.67 mmol), linoleic acid (1.2 eqv., 224.0 mg, 0.8 mmol), DMAP (1.2 eqv., 122 mg, 0.8 mmol) and EDC (1.2 eqv., 155 mg, 0.8 mmol) were mixed and dissolved in about 2 mL ultra-dry DCM. The reaction solution was stirred and heated at 45 ℃ overnight on the magnetic hotplate stirrer. The reaction progress was monitored using TLC. Upon completion of the reaction, the reaction mixture was cooled and subsequently washed with 5% w/w citric acid, saturated aqueous NaHCO_3_, and brine. The organic layer was dried over anhydrous sodium sulfate and then evaporated under rotary evaporation. The product was further subjected to purification using column chromatography on silica gel in DCM: MeOH (50:1) as the mobile phase to obtain Car-LA (130.6 mg, 47.3%). The purified product was subsequently subjected to structural analysis using ^1^H NMR.

**Synthesis of TSPBA**

**
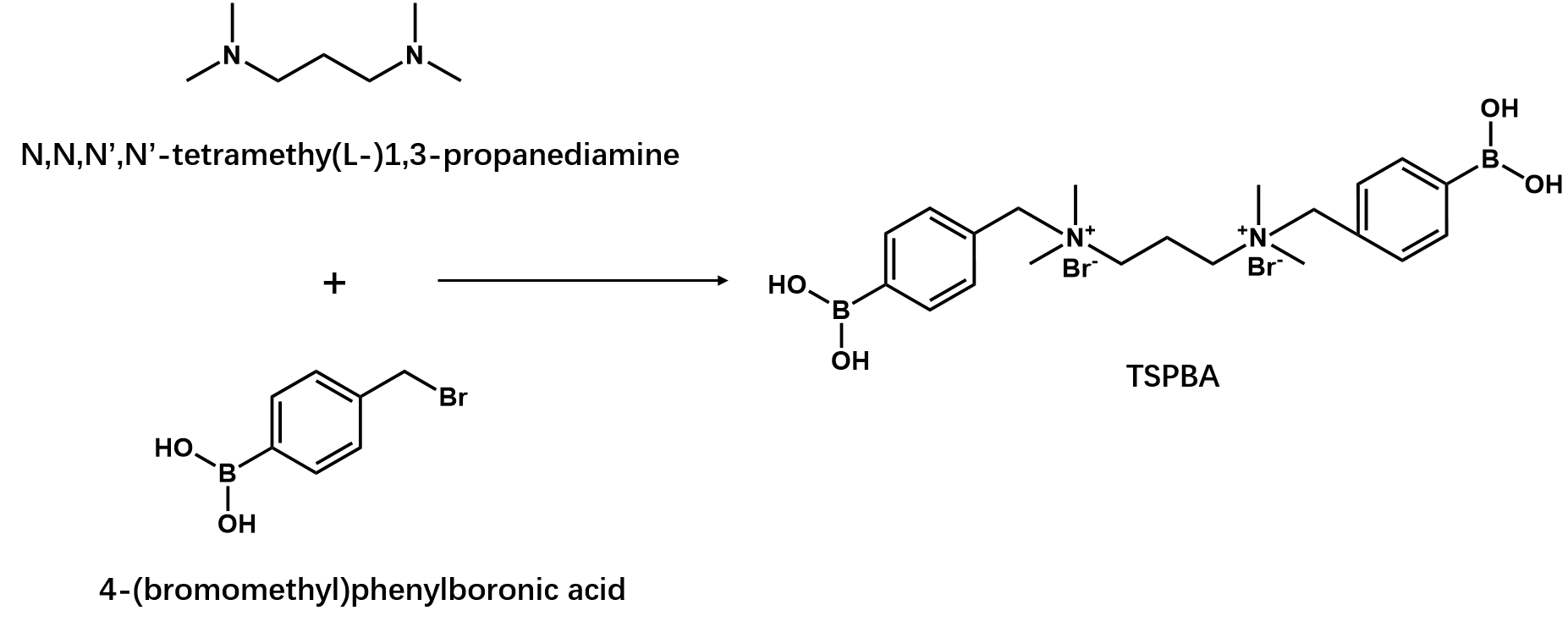
**

N,N,N’,N’-tetramethy(L-)1,3-propanediamine (TMPDA, 1.0 eqv., 100 mg, 0.75 mmol) and 4-(bromomethyl)phenylboronic acid (3.0 eqv., 500 mg, 2.3 mmol) were dissolved in 10 mL of anhydrous DMF and stirred at 60 °C overnight. The mixture was subsequently poured into 100 mL tetrahydrofuran (THF), filtered and washed with THF three times. The precipitate was then dissolved in deionized water, flash-frozen in liquid nitrogen and lyophilized under vacuum for 48 h to obtain purified TSPBA.


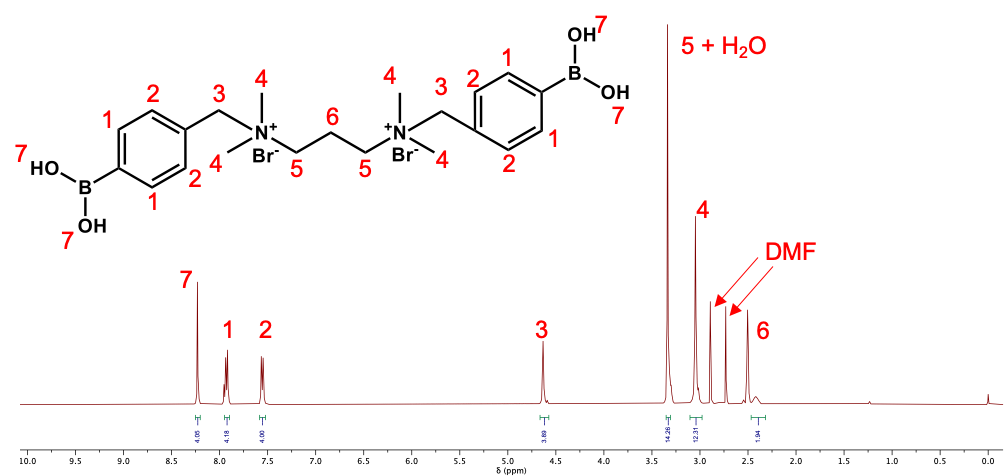


**Fig.S1. ^1^H NMR spectrum of TSPBA.**

1H NMR (400 MHz, d-DMSO) δ 8.23 (s, 4H), 7.94-7.92 (d, J = 8.0 Hz, 4H), 7.57-7.55 (d, J = 8.0 Hz, 4H), 4.63 (s, 4H), 3.34 (s, 4H), 3.04 (s, 12H), 2.43-2.33 (m, 2H)


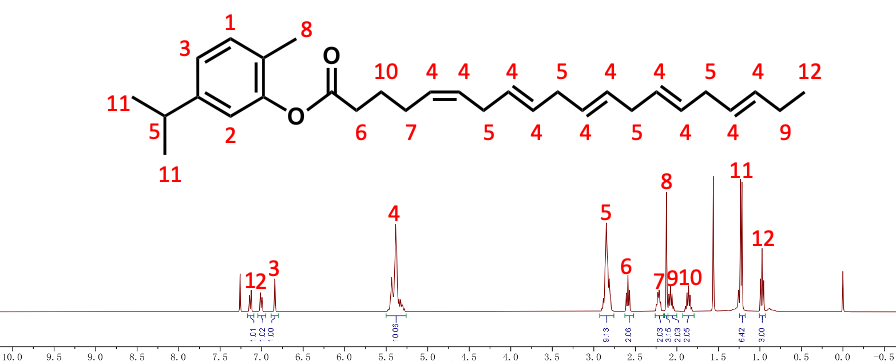


**Fig.S2. ^1^H NMR spectrum of the Car-EPA.**

^1^H NMR (400 MHz, d-CDCl3) δ 7.15-7.13 (d, J = 8.0 Hz, 1H), 7.02-7.00 (d, J = 8.0 Hz, 1H), 6.84 (s, 1H), 5.44-5.28 (m, 10H), 2.90-2.80 (m, 9H), 2.61-2.57 (t, J = 8.0 Hz, 2H), 2.23-2.22 (q, J = 4.0 Hz, 2H), 2.12 (s, 3H), 2.10-2.04 (m, 2H), 1.90-1.82 (m, 2H), 1.25-1.21 (t, J = 8.0 Hz, 6H), 0.99-0.95 (t, J = 8.0 Hz, 3H)


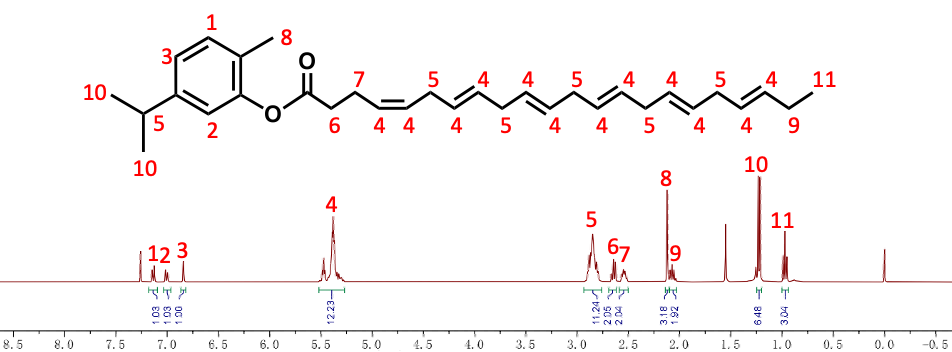


**Fig.S3. ^1^H NMR spectrum of the Car-DHA.**

^1^H NMR (400 MHz, d-CDCl3) δ 7.15-7.13 (d, J = 8.0 Hz, 1H), 7.02-7.00 (d, J = 8.0 Hz, 1H), 6.84 (s, 1H), 5.51-5.28 (m, 12H), 2.90-2.80 (m, 11H), 2.66-2.62 (t, J = 8.0 Hz, 2H), 2.56-2.52 (q, J = 4.0 Hz, 2H), 2.12 (s, 3H), 2.10-2.04 (m, 2H), 1.25-1.21 (t, J = 8.0 Hz, 6H), 0.99-0.95 (t, J = 8.0 Hz, 3H)


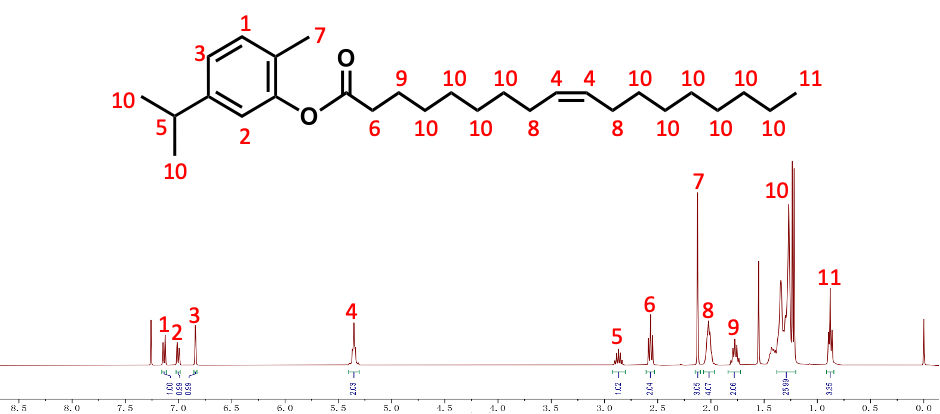


**Fig.S4. ^1^H NMR spectrum of the Car-OA.**

^1^H NMR (400 MHz, d-CDCl3) δ 7.15-7.13 (d, J = 8.0 Hz, 1H), 7.02-7.00 (d, J = 8.0 Hz, 1H), 6.84 (s, 1H), 5.40-5.30 (m, 2H), 2.92-2.82 (m, 1H), 2.59-2.55 (t, J = 8.0 Hz, 2H), 2.12 (s, 3H), 2.03-1.99 (m, 4H), 1.81-1.74 (m, 2H), 1.37-1.22 (m, 26H), 0.90-0.86 (t, J = 8.0 Hz, 3H)


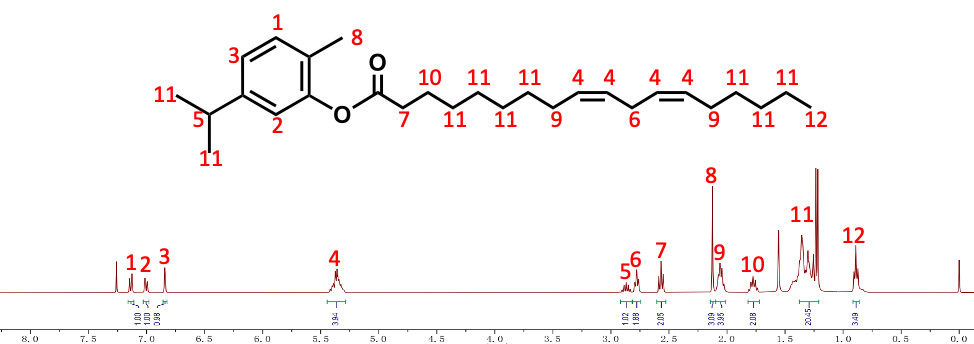


**Fig.S5. ^1^H NMR spectrum of the Car-LA.**

^1^H NMR (400 MHz, d-CDCl3) δ 7.15-7.13 (d, J = 8.0 Hz, 1H), 7.02-7.00 (d, J = 8.0 Hz, 1H), 6.84 (s, 1H), 5.42-5.30 (m, 4H), 2.90-2.83 (m, 1H), 2.80-2.76 (t, J = 8.0 Hz, 2H), 2.59-2.55 (t, J = 8.0 Hz, 2H), 2.13 (s, 3H), 2.07-2.03 (m, 4H), 1.81-1.74 (m, 2H), 1.37-1.22 (m, 20H), 0.91-0.87 (t, J = 8.0 Hz, 3H)


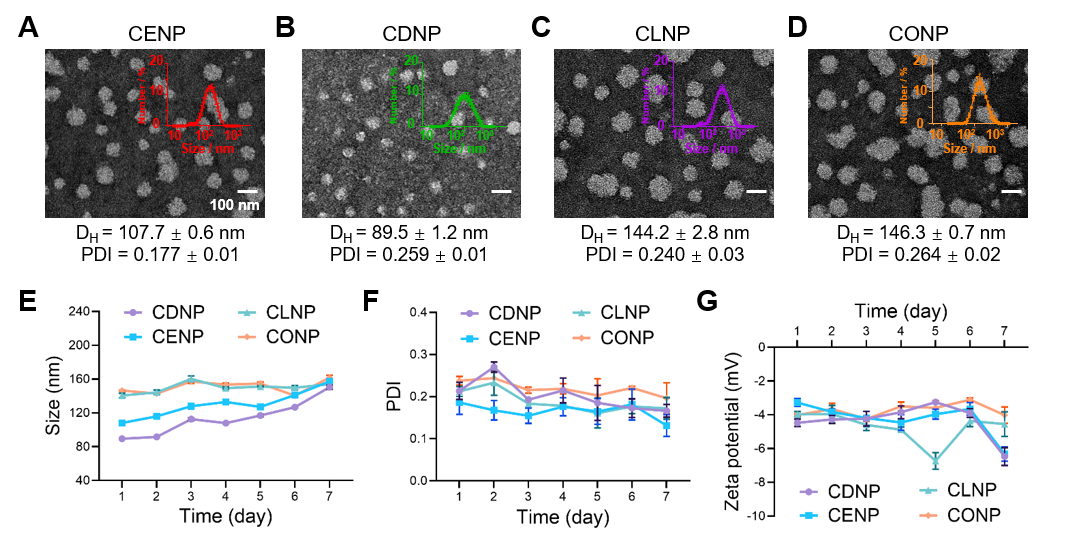


**Fig.S6. Characterization of carvacrol prodrug.** (A-D) Representative TEM image and corresponding size distribution of carvacrol prodrug. Scale bar: 100 nm. (E) Particle size stability of carvacrol prodrug monitored continuously at 37 °C over 7 days. (F) Polydispersity index (PDI) of carvacrol prodrug monitored continuously at 37 °C over 7 days. (G) Zeta potential of carvacrol prodrug monitored continuously at 37 °C over 7 days. Data are expressed as mean ± SD (n = 3).

**
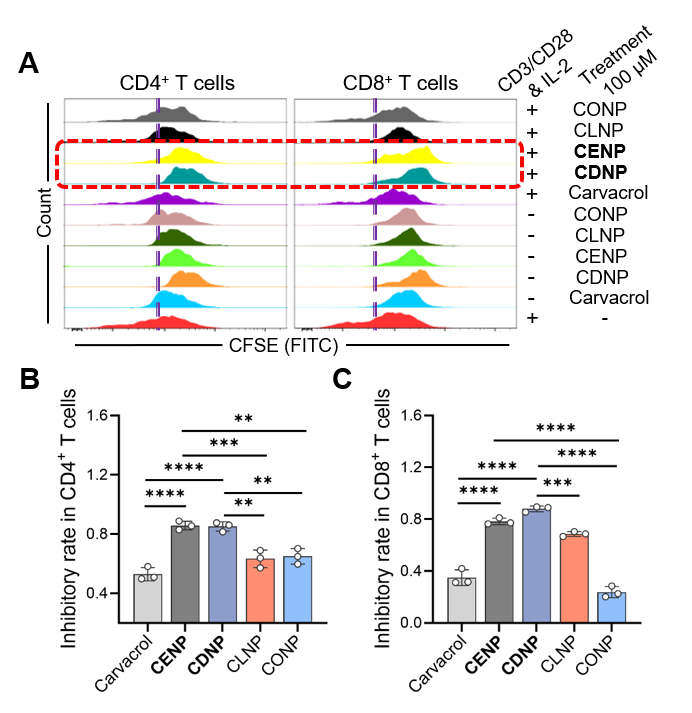
**

**Fig.S7. Effect of carvacrol prodrug on T-cell proliferation in vitro.** (A) Representative proliferation profiles of T cells treated with different carvacrol prodrug formulations. (B) Inhibitory effect of carvacrol prodrug on CD4^+^ T-cell proliferation. (C) Inhibitory effect of carvacrol prodrug on CD8^+^ T-cell proliferation. Data are expressed as mean ± SD (n = 3). Statistical significance was assessed by one-way ANOVA with Tukey's post hoc test (B and C): ***p* < 0.01; ****p* < 0.001; and *****p* < 0.0001.

**
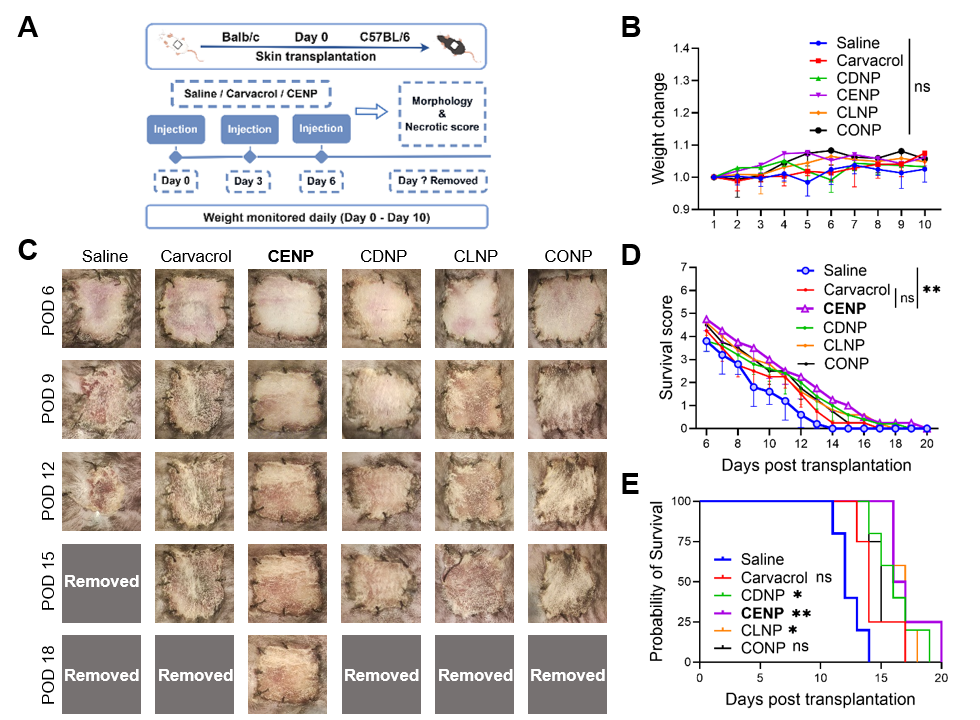
**

**Fig.S8. Application of carvacrol prodrug in allogeneic skin transplantation.** (A) Schematic diagram of carvacrol prodrug treatment and experimental monitoring protocol following allogeneic skin transplantation. (B) Dynamic changes in murine body weight during the intervention period. (C) Representative gross morphology of skin allografts in different treatment groups. (D) Quantitative analysis of skin allograft survival scores from POD6 to graft removal. (E) Graft survival rate curves over 20 days post-transplantation. Data are expressed as mean ± SD (n = 4-5). Statistical significance was assessed by two-way ANOVA followed by Tukey's post hoc (B and D) or log-rank (Mantel−Cox) test (E): **p* < 0.05; ***p* < 0.01; and ns, not significant.


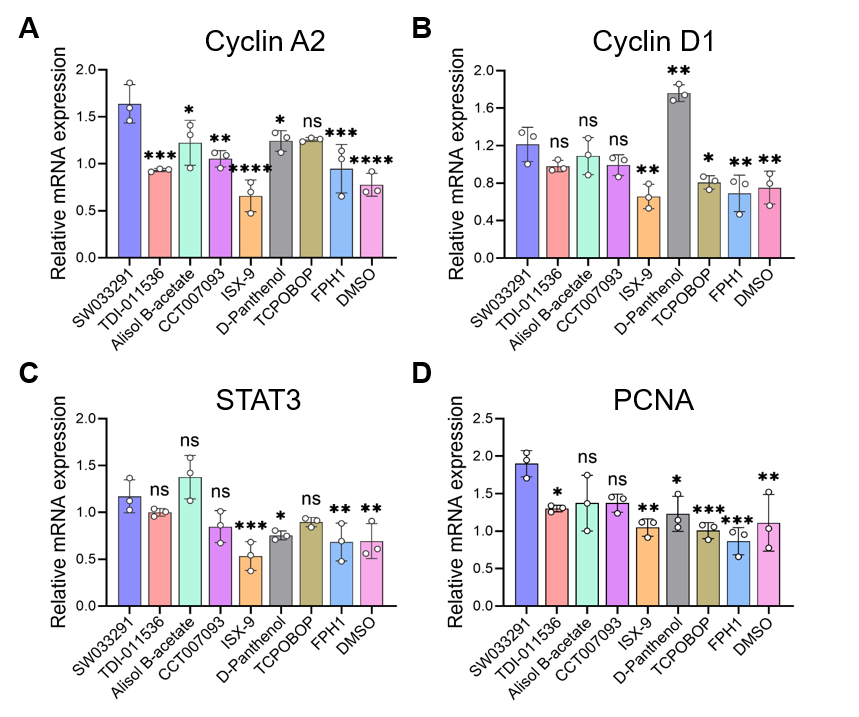


**Fig.S9. Evaluation of pro-regenerative effects of candidate therapeutic molecules.** NIH-3T3 cells were treated with 200 nM candidate drug for 48 h. Relative expression levels of Cyclin A2, Cyclin D1, STAT3, and PCNA were shown. Data are expressed as mean ± SD (n = 3). Statistical significance was assessed by one-way ANOVA with Tukey's post hoc test. All comparisons were made against the SW033291 group: (A to D): **p* < 0.05; ***p* < 0.01; ****p* < 0.001; *****p* < 0.0001; and ns, not significant.


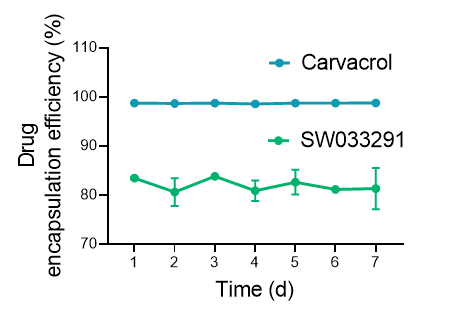


**Fig.S10.** Encapsulation efficiency of carvacrol and SW033291 in CSNP determined by HPLC. Data are expressed as mean ± SD (n = 3).


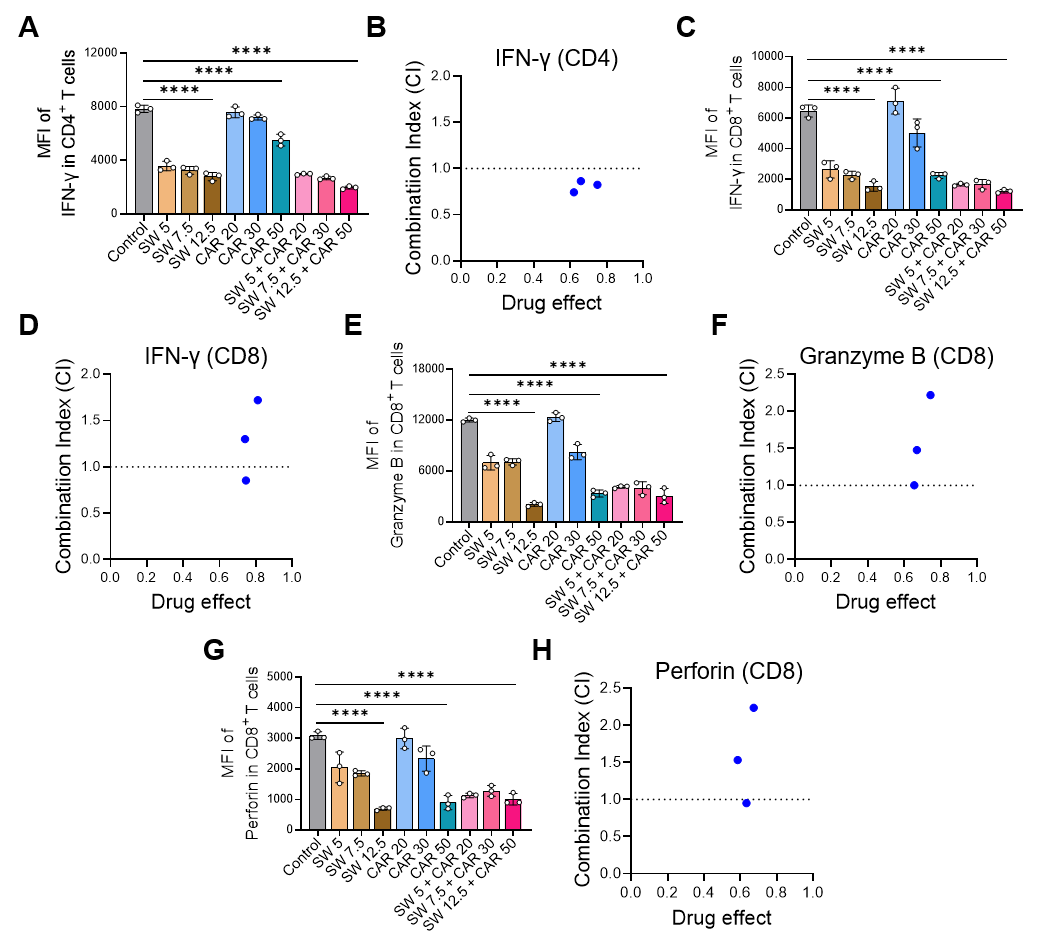


**Fig.S11. Evaluation of the immunosuppressive interactions between carvacrol and SW033291 in T cells.** (A) Mean fluorescence intensity (MFI) of IFN-γ expression in activated CD4⁺ T cells treated with carvacrol (20, 30, 50 μM) and SW033291 (5, 7.5, 12.5 μM), alone or in combination. (B) Combination index (CI) analysis of carvacrol and SW033291 for inhibition of IFN-γ expression in CD4⁺ T cells. (C) MFI of IFN-γ expression in activated CD8⁺ T cells treated with carvacrol (20, 30, 50 μM) and SW033291 (5, 7.5, 12.5 μM), alone or in combination. (D) CI analysis of carvacrol and SW033291 for inhibition of IFN-γ expression in CD8⁺ T cells. (E) MFI of granzyme B expression in activated CD8⁺ T cells treated with carvacrol (20, 30, 50 μM) and SW033291 (5, 7.5, 12.5 μM), alone or in combination. (F) CI analysis of carvacrol and SW033291 for inhibition of granzyme B expression in CD8⁺ T cells. (G) MFI of perforin expression in activated CD8⁺ T cells treated with carvacrol (20, 30, 50 μM) and SW033291 (5, 7.5, 12.5 μM), alone or in combination. (H) CI analysis of carvacrol and SW033291 for inhibition of perforin expression in CD8⁺ T cells. Data are expressed as mean ± SD (n = 3). Statistical significance was assessed by one-way ANOVA with Tukey's post hoc test: *****p* < 0.0001.

**
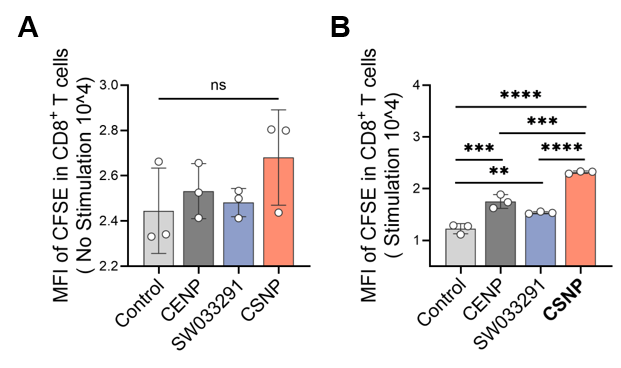
**

**Fig.S12. Effects of CENP, CSNP, and SW033291 on CD8^+^** **T cells proliferation in vitro.** (A) Mean fluorescence intensity (MFI) of CFSE in CD8^+^ T cells treated with CENP, CSNP, or SW033291 under conditions without IL-2 and CD3/CD28 stimulation. (B) MFI of CFSE in CD8^+^ T cells treated with the same compounds under conditions with IL-2 and CD3/CD28 stimulation. Data are expressed as mean ± SD (n = 3). Statistical significance was assessed by one-way ANOVA with Tukey's post hoc test (A and B): ***p* < 0.01; ****p* < 0.001; *****p* < 0.0001; and ns, not significant.


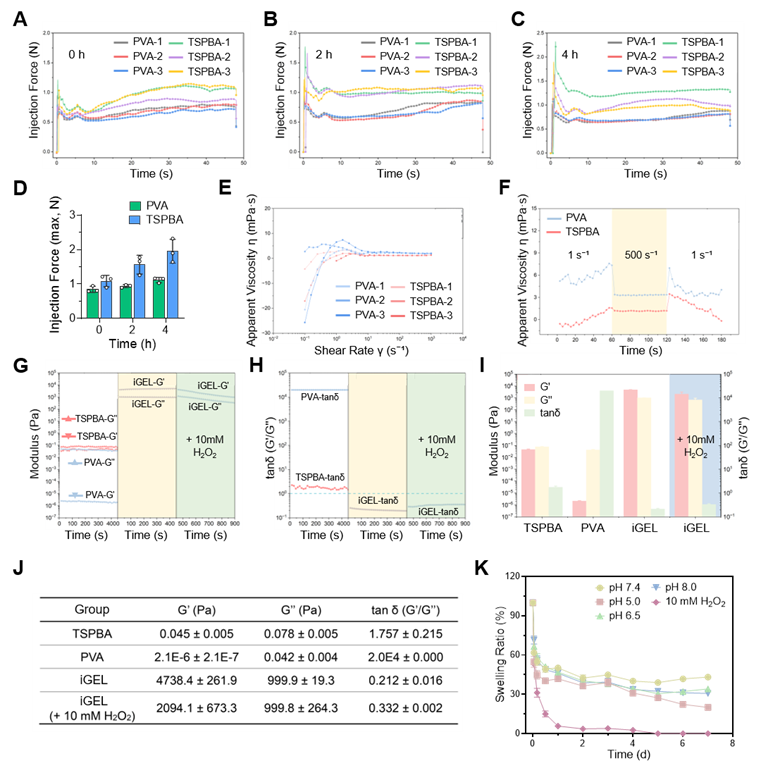


**Fig.S13.** **Evaluation of the physicochemical properties of iGEL.** (A-C) Injection force profiles of PVA and TSPBA formulations over time. (D) Maximum injection force required for PVA and TSPBA at different time points. (E) Shear viscosity curves of PVA and TSPBA precursor solutions. (F) Thixotropic recovery behavior of PVA and TSPBA subjected to a three-step shear cycle. (G) Dynamic time-sweep rheology (1 Hz, 1% strain) for PVA, TSPBA, and iGEL. (H) Evolution of tanδ (G"/G') for PVA, TSPBA, and iGEL over time under dynamic rheological testing. (I) Comparison of key rheological parameters (G', G", tan δ) for PVA, TSPBA, and iGEL. (J) Summary table of rheological data (G', G", tan δ) for PVA, TSPBA, and iGEL. (K) Swelling ratio of iGEL hydrogels over 7 days under various pH conditions and 10 mM H_2_O_2_. Data are expressed as mean ± SD (n = 3).


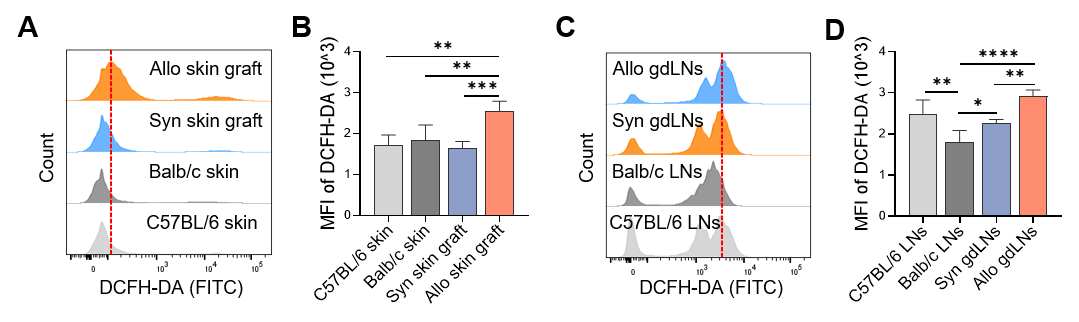


**Fig.S14. Quantitative detection of reactive oxygen species levels on POD4.** (A) Flow cytometry histograms of ROS levels in skin tissues on POD4. Allo skin graft: Skin graft in the BALB/c-to-C57BL/6 skin transplantation model. Syn skin graft: Skin graft in the C57BL/6-to-C57BL/6 skin transplantation model. (B) Statistical analysis of the mean fluorescence intensity (MFI) of ROS in skin tissues on POD4. (C) Flow cytometry histograms of ROS levels in lymph nodes on POD4. Allo gdLNs: Graft draining lymph nodes in the BALB/c-to-C57BL/6 skin transplantation model. Syn gdLNs: Graft draining lymph nodes in the C57BL/6-to-C57BL/6 skin transplantation model. (D) Statistical analysis of the MFI of ROS in lymph nodes on POD4. Data are expressed as mean ± SD (n = 5). Statistical significance was assessed by one-way ANOVA with Tukey's post hoc test: **p* < 0.05; ***p* < 0.01; ****p* < 0.001; and *****p* < 0.0001.


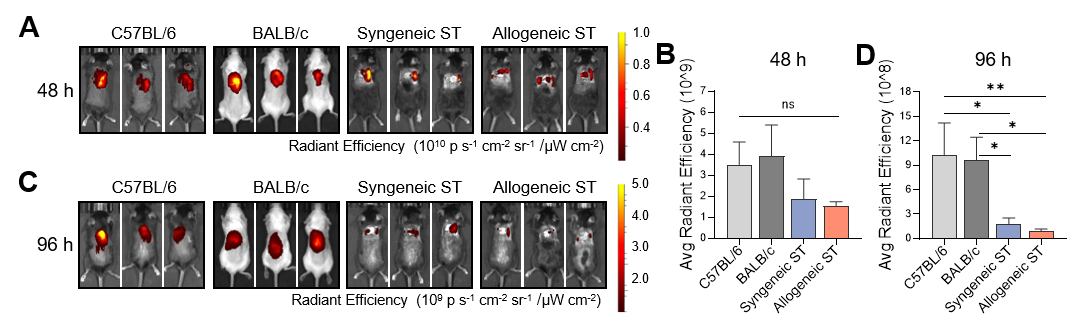


**Fig.S15. Degradation of hydrogel under physiological and pathological reactive oxygen species conditions.** (A) Representative fluorescence images showing iGEL retention at 48 hours post-injection and hydrogel formation. (B) Quantitative analysis of fluorescence signal intensity at 48 hours post-hydrogel injection. (C) Representative fluorescence images showing iGEL retention at 96 hours post-injection and hydrogel formation. (D) Quantitative analysis of fluorescence signal intensity at 96 hours post-hydrogel injection. Syngeneic ST: C57BL/6-to-C57BL/6 skin transplantation model. Allogeneic ST: BALB/c-to-C57BL/6 skin transplantation model. Data are expressed as mean ± SD (n = 3). Statistical significance was assessed by one-way ANOVA with Tukey's post hoc test: **p* < 0.05; ***p* < 0.01; and ns, not significant.


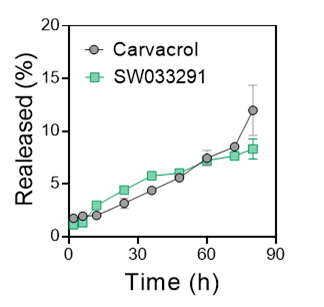


**Fig.S16.** Cumulative release profiles of SW033291 and carvacrol in T lymphocyte-conditioned media. Data are expressed as mean ± SD (n = 3).

**
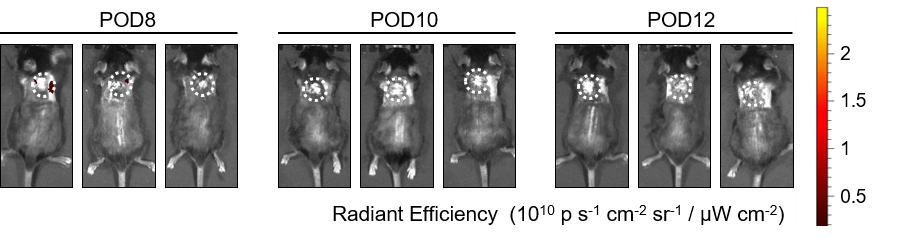
**

**Fig.S17.** Representative fluorescence images showing iGEL retention around the allograft on postoperative day 8 (POD8), POD10 and POD12.

**
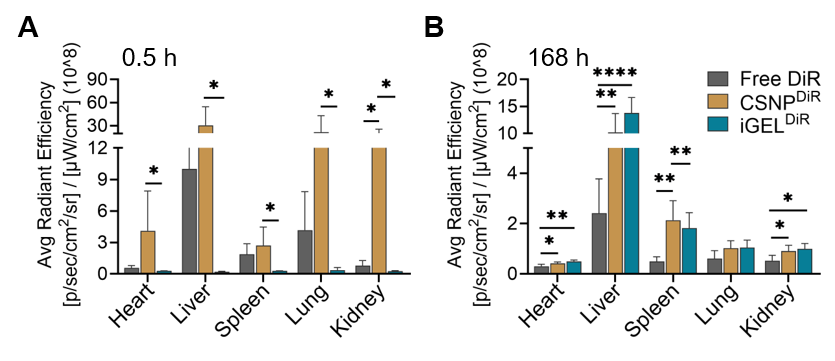
**

**Fig.S18.** Quantitative analysis of fluorescence signal intensity in ex vivo heart, liver, spleen, lung, and kidney tissues at 0.5 h and 168 h post-treatment with DiR-labeled nanoparticles or hydrogel. Data are expressed as mean ± SD (n = 4-5). Statistical significance was assessed by one-way ANOVA with Tukey's post hoc test: **p* < 0.05; ***p* < 0.01; and *****p* < 0.0001.

**
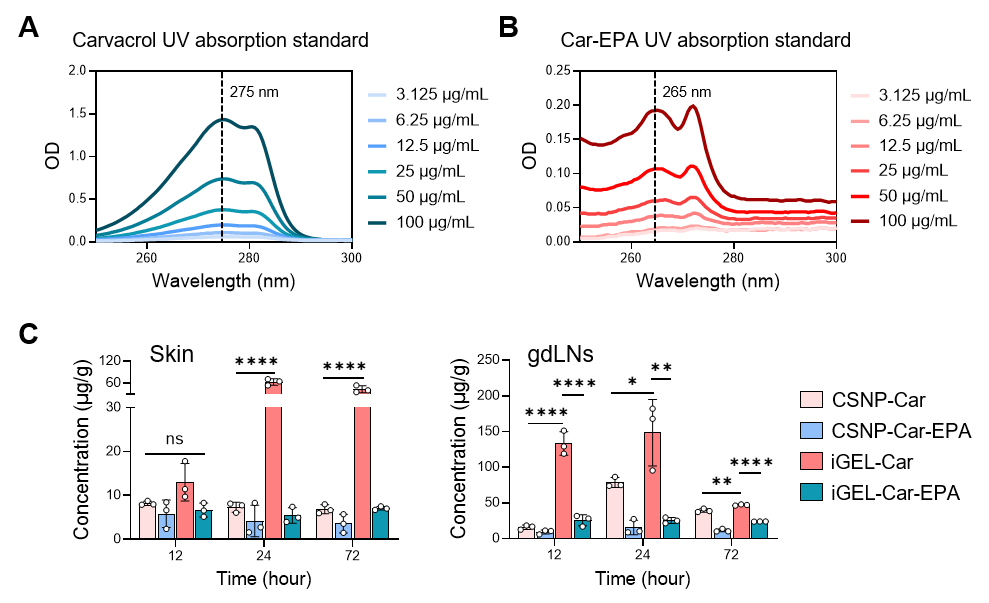
**

**Fig.S19.** **Evaluation of drug accumulation level within tissues.** (A) Line graph illustrating UV absorption spectra of carvacrol at concentrations ranging from 3.125 to 100 µg/mL. (B) Line graph depicting UV absorption spectra of Car-EPA at concentrations from 3.125 to 100 µg/mL. (C) Time-dependent concentrations (µg/g) of carvacrol and Car-EPA in skin allografts and gdLNs at 12, 24, and 72 hours post-treatment. Data are expressed as mean ± SD (n = 3). Statistical significance was assessed by one-way ANOVA with Tukey's post hoc test: **p* < 0.05; ***p* < 0.01; *****p* < 0.0001; and ns, not significant.

**
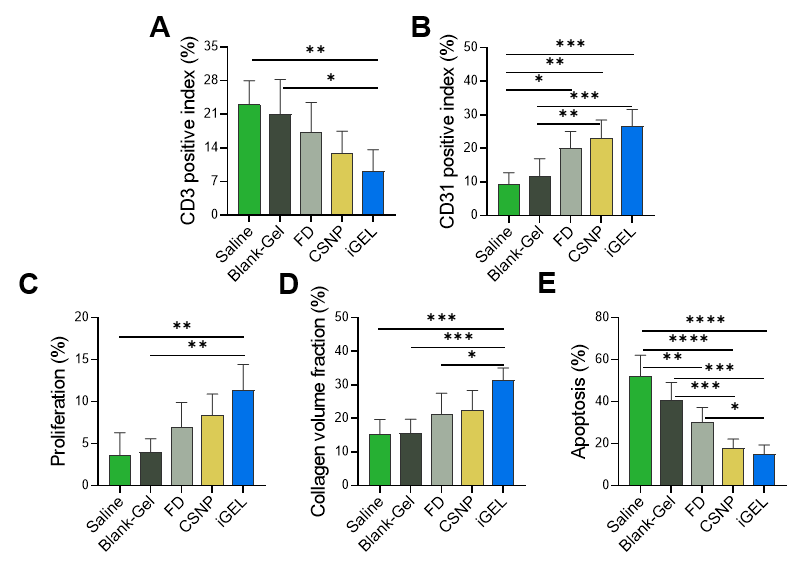
**

**Fig.S20.** Quantitative analysis of CD3 (A), CD31 (B), Cyclin D1 (C), Masson (D), and TUNEL (E) staining, quantified by ImageJ software. Data are expressed as mean ± SD (n = 5). Statistical significance was assessed by one-way ANOVA with Tukey's post hoc test: **p* < 0.05; ***p* < 0.01; ****p* < 0.001; and *****p* < 0.0001.

**
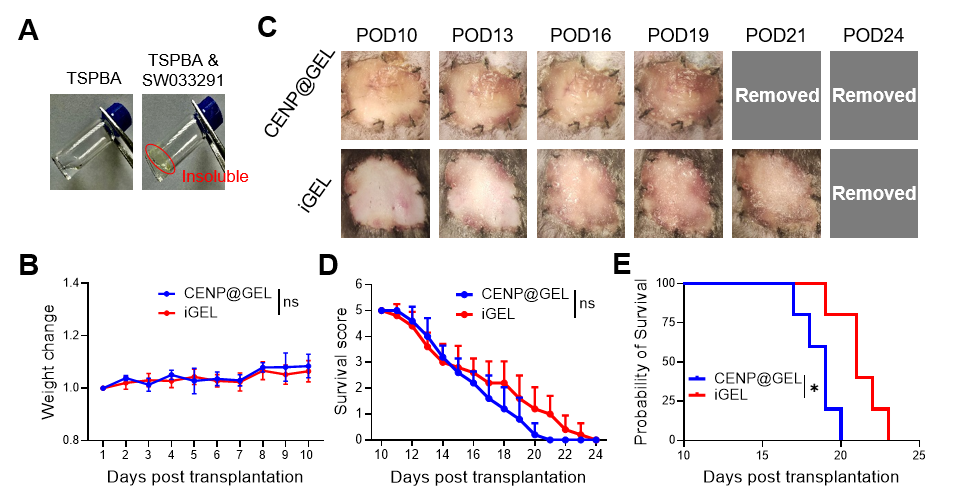
**

**Fig. S21. Application of iGEL or CENP@GEL in allogeneic skin transplantation.** (A) Photographs of TSPBA solution and TSPBA solution containing SW033291 (with SW033291 insoluble). (B) Body weight changes in mice during treatment. (C) Representative gross morphology of skin allografts in different treatment groups. (D) Quantitative analysis of skin allograft survival scores from POD10 until graft loss. (E) Kaplan–Meier survival curves of grafts over 23 days post-transplantation. Data are expressed as mean ± SD (n = 5). Statistical significance was assessed by two-way ANOVA followed by Tukey's post hoc (B and D) or log-rank (Mantel−Cox) test (E): **p* < 0.05; and ns, not significant.

**
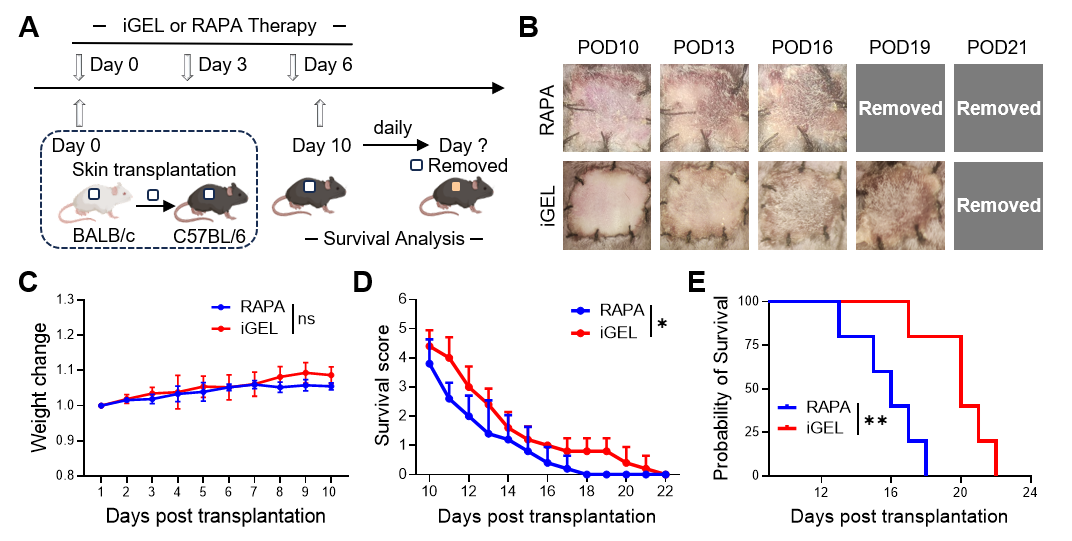
**

**Fig.S22.** **Application of iGEL or Rapamycin (RAPA) in allogeneic skin transplantation.** (A) Schematic of the iGEL treatment protocol and monitoring timeline after skin transplantation. RAPA: Rapamycin. (B) Representative gross morphology of skin allografts in different treatment groups. (C) Body weight changes in mice during treatment. (D) Quantitative analysis of skin allograft survival scores from POD10 until graft loss. (E) Kaplan–Meier survival curves of grafts over 22 days post-transplantation. Data are expressed as mean ± SD (n = 5). Statistical significance was assessed by two-way ANOVA followed by Tukey's post hoc (C and D) or log-rank (Mantel−Cox) test (E): **p* < 0.05; ***p* < 0.01; and ns, not significant.

**
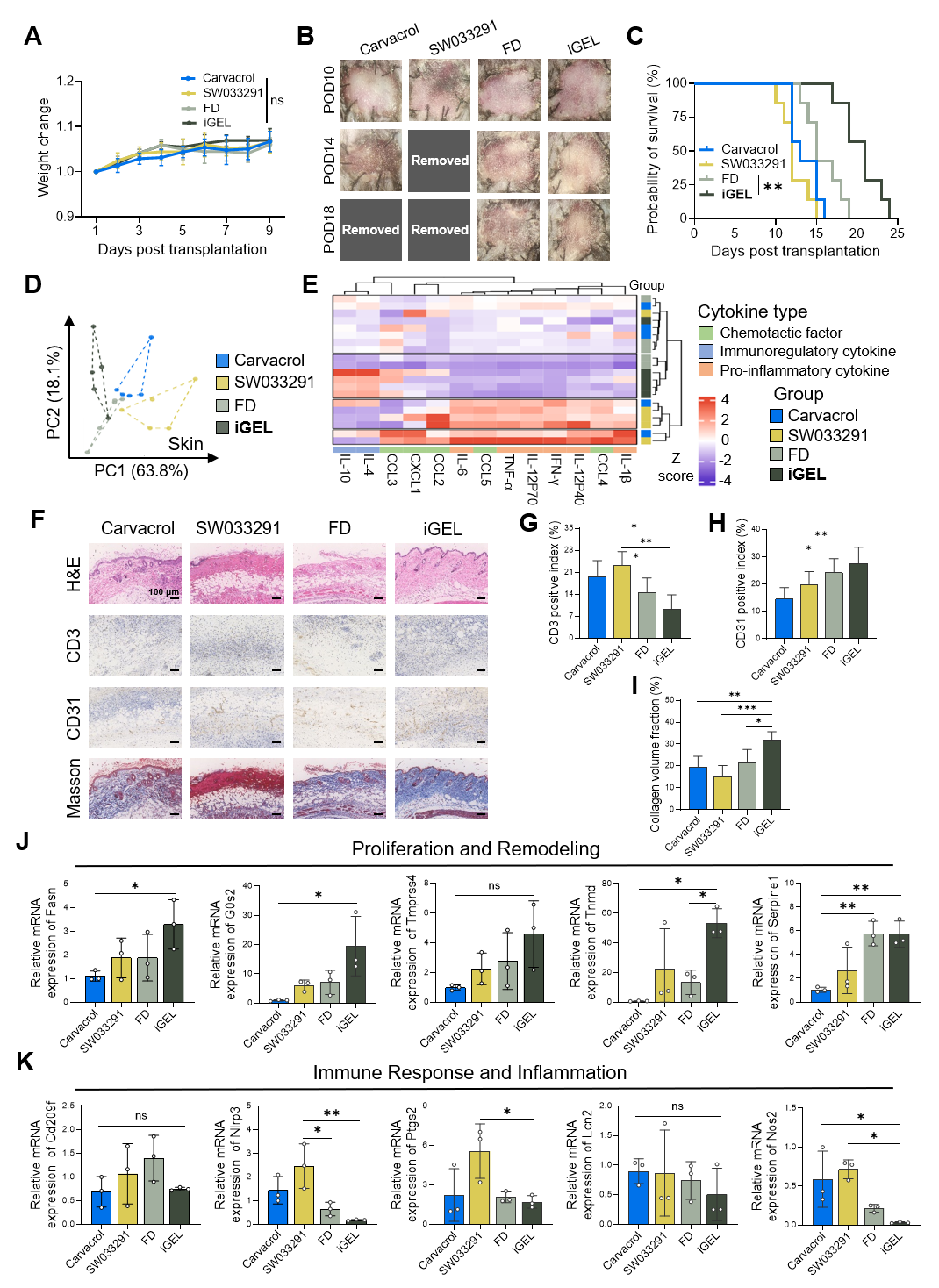
**

**Fig.S23. Therapeutic effects of monotherapy or iGEL in allogeneic skin transplantation.** (A) Body weight changes in mice during treatment. (B) Representative gross morphology of allografts in different treatment groups. (C) Kaplan–Meier survival curves of grafts over 24 days post-transplantation. (D) PCA of cytokine profiles from skin allografts (13 cytokines). (E) Hierarchical clustering heatmaps of cytokine expression in skin allografts. (F) Histopathological evaluation on POD8: H&E staining, immunohistochemistry (CD3^+^ T cells, CD31^+^ vasculature), Masson's trichrome staining (blue: collagen). Scale bar: 100 μm. Quantitative analysis of CD3 (G), CD31 (H), and Masson (I) staining determined by ImageJ software. (J) Relative mRNA expression levels of proliferation and remodeling genes. (K) Relative mRNA expression levels of immune response and inflammation genes. Data are expressed as mean ± SD (n = 7 in A and C; n = 5 in D to I; and n = 3 in J and K). Statistical significance was assessed by two-way ANOVA followed by Tukey's post hoc (A), log-rank (Mantel−Cox) test (C), and one-way ANOVA with Tukey's post hoc test (G to K): **p* < 0.05; ***p* < 0.01; ****p* < 0.001; and ns, not significant.

**
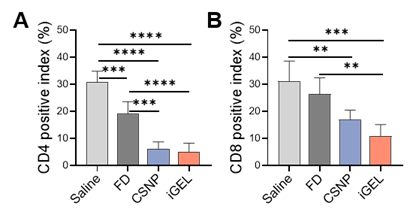
**

**Fig.S24.** Quantitative analysis of CD4 (A) and CD8 (B) staining determined by ImageJ software. Data are expressed as mean ± SD (n = 5). Statistical significance was assessed by one-way ANOVA with Tukey's post hoc test: ***p* < 0.01; ****p* < 0.001; *****p* < 0.0001.

**
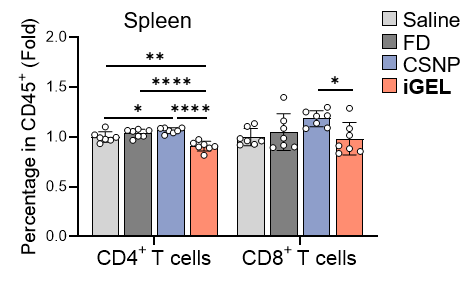
**

**Fig.S25.** Flow cytometric analysis of CD4^+^ and CD8^+^ T cell subsets in CD45^+^ populations from spleen. Data are expressed as mean ± SD (n = 7). Statistical significance was assessed by one-way ANOVA with Tukey's post hoc test: **p* < 0.05; ***p* < 0.01; and *****p* < 0.0001.

**
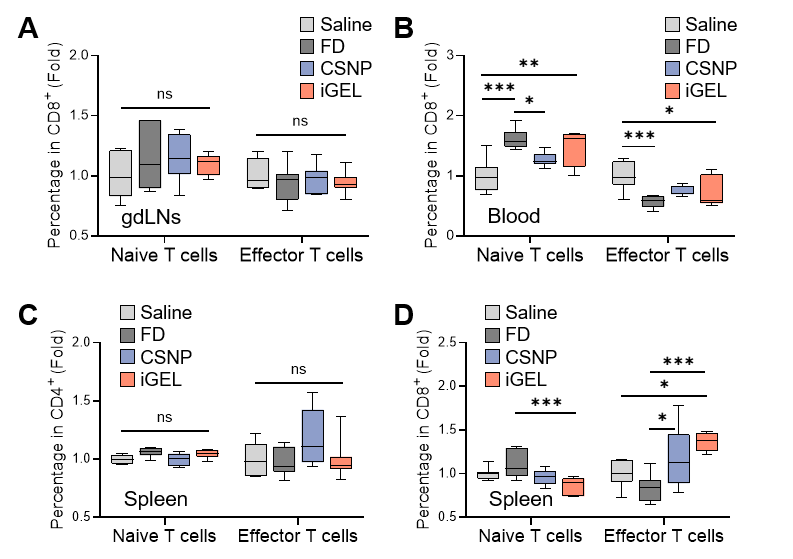
**

**Fig.S26. Flow cytometric analysis of naive and effector T cell subpopulations.** (A) Naïve T cell (CD44⁻CD62L⁺) and effector T cell (CD44⁺CD62L⁻) proportions within CD8^+^ populations from graft draining lymph nodes (gdLNs). (B) Naïve T cell and effector T cell proportions within CD8^+^ populations from peripheral blood. (C, D) Naïve T cell and effector T cell proportions within CD4^+^ or CD8^+^ populations from spleen. Data are expressed as mean ± SD (n = 7). Statistical significance was assessed by one-way ANOVA with Tukey's post hoc test: **p* < 0.05; ***p* < 0.01; ****p* < 0.001; and ns, not significant.


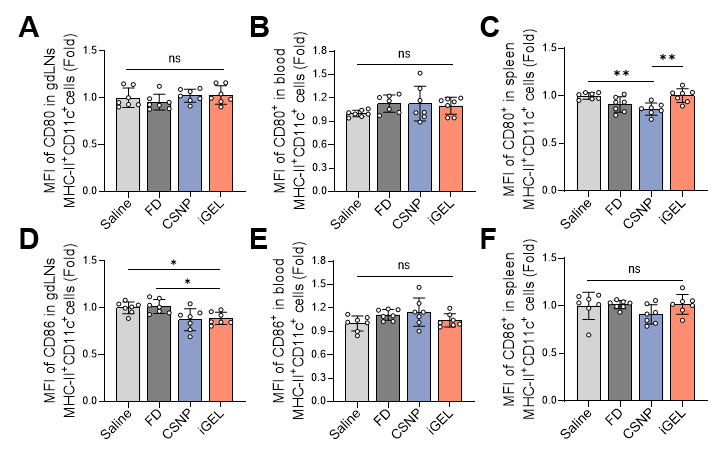


**Fig.S27.** **Analysis of dendritic cell maturation.** (A-C) Mean fluorescence intensity (MFI) of CD80 in dendritic cells (MHC-II⁺CD11c⁺) from graft-draining lymph nodes (gdLNs), peripheral blood, and spleen. (D-F) MFI of CD86 in dendritic cells from gdLNs, peripheral blood, and spleen. Data are expressed as mean ± SD (n = 7). Statistical significance was assessed by one-way ANOVA with Tukey's post hoc test: **p* < 0.05; ***p* < 0.01; and ns, not significant.

**
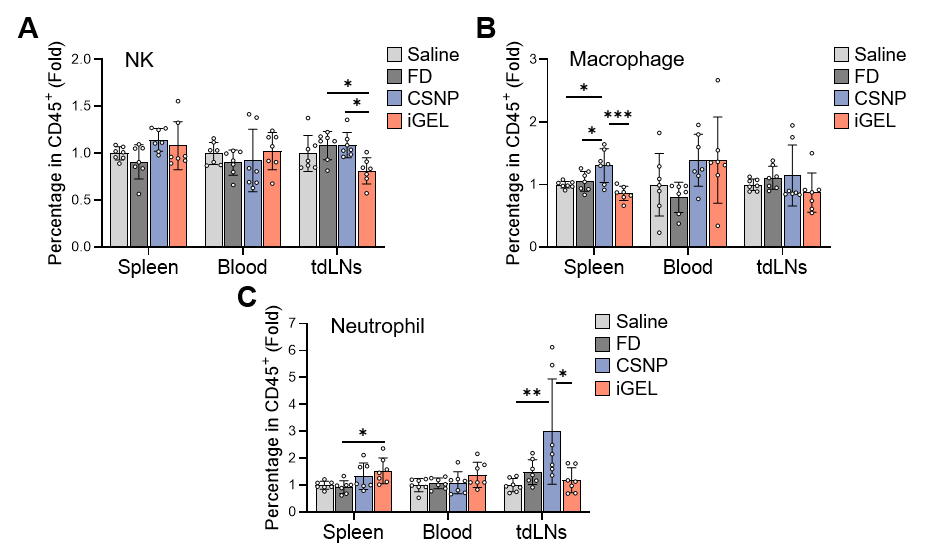
**

**Fig.S28. Flow cytometric analysis of NK cells, macrophages, and neutrophils subpopulations.** (A) Proportion of NK cells (CD3^-^NK1.1^+^) among CD45^+^ cells in spleen, peripheral blood, and gdLNs. (B) Proportion of macrophage (CD11b^+^F4/80^+^) among CD45^+^ cells in spleen, peripheral blood, and gdLNs. (C) Proportion of neutrophil (CD11b^+^Ly6G^+^) among CD45^+^ cells in spleen, peripheral blood, and gdLNs. Data are expressed as mean ± SD (n = 7). Statistical significance was assessed by one-way ANOVA with Tukey's post hoc test: **p* < 0.05; ***p* < 0.01; and ****p* < 0.001.


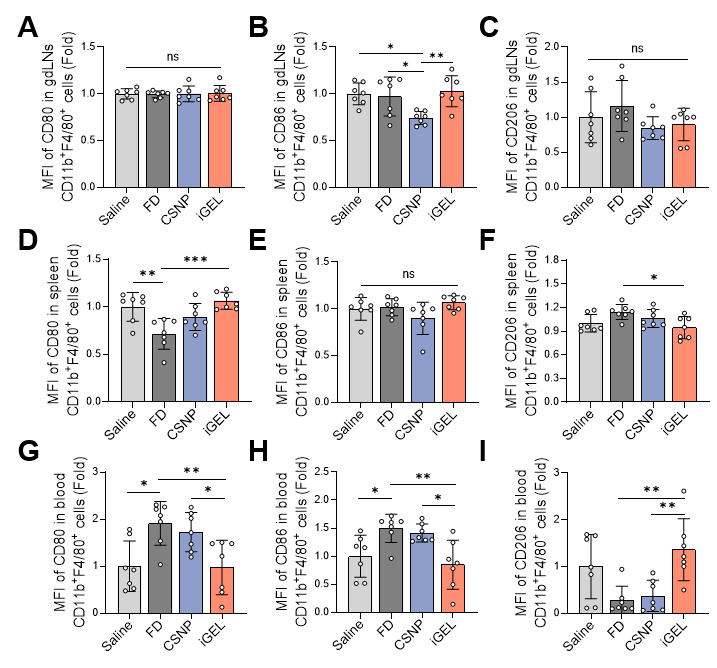


**Fig.S29.** **Analysis of macrophage polarization.** (A-C) Mean fluorescence intensity (MFI) of CD80, CD86, and CD206 in macrophages (CD11b^+^F4/80⁺) from graft-draining lymph nodes (gdLNs). (D-F) MFI of CD80, CD86, and CD206 in macrophages from spleen. (G-I) MFI of CD80, CD86, and CD206 in macrophages from peripheral blood. Data are expressed as mean ± SD (n = 7). Statistical significance was assessed by one-way ANOVA with Tukey's post hoc test: **p* < 0.05; ***p* < 0.01; ****p* < 0.001; and ns, not significant.


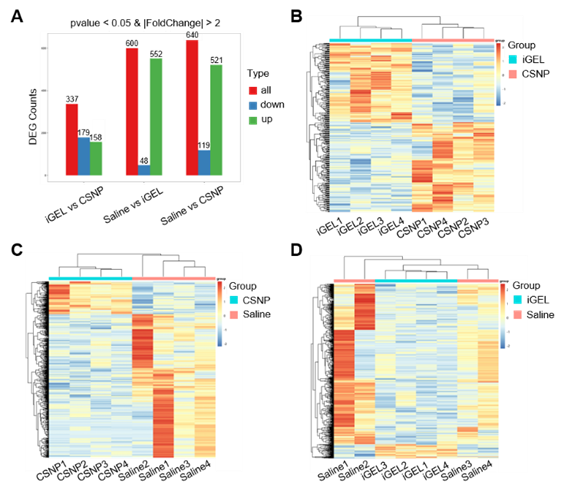


**Fig.S30.** **Analysis of differentially expressed genes (DEGs).** (A) Statistical chart of DEGs counts across various comparison groups. (B-D) Differentially expressed gene clustering heatmap.

**
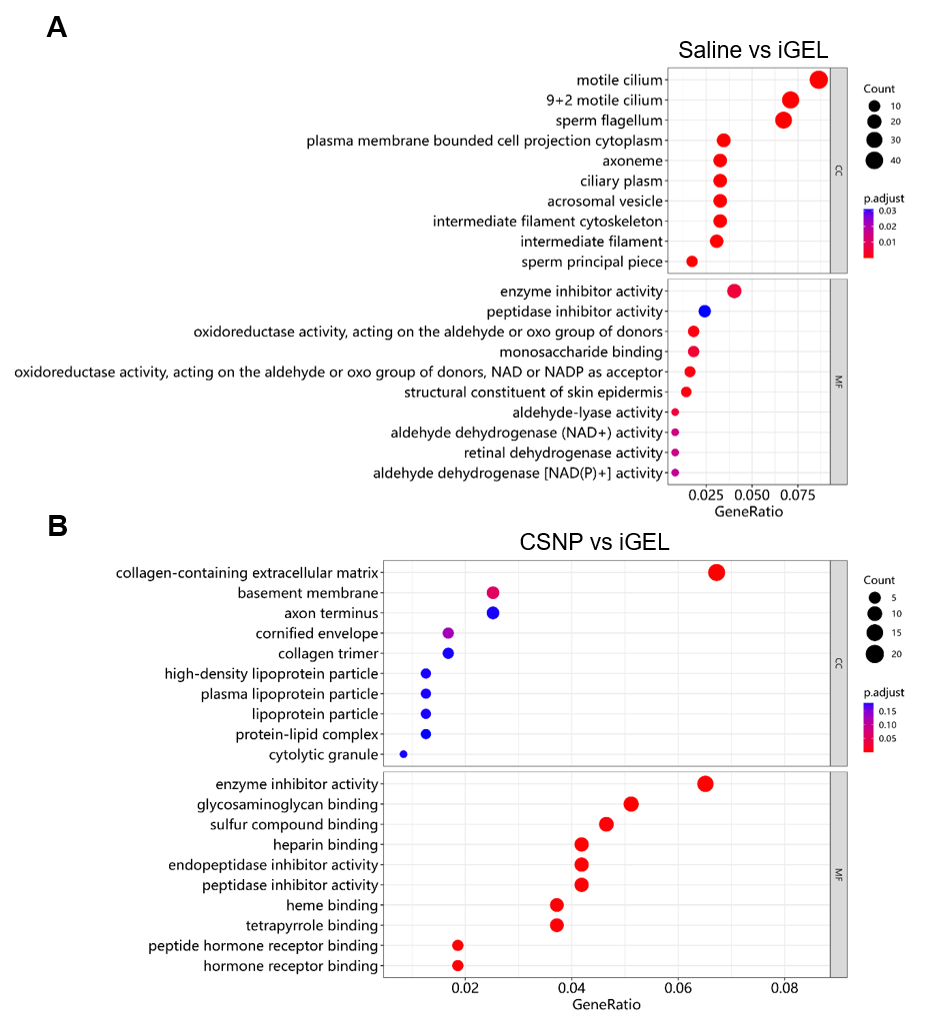
**

**Fig.S31.** **GO enrichment analysis of differentially expressed genes.** (A) GO enrichment analysis of differentially expressed genes between Saline and iGEL groups. (B) GO enrichment analysis of differentially expressed genes between CSNP and iGEL groups. CC: Cellular Component; MF: Molecular Function.

**
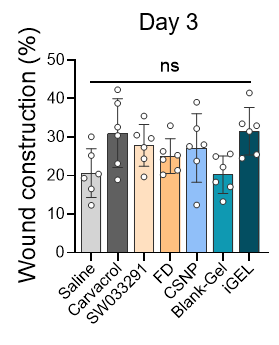
**

**Fig.S32.** Statistical analysis of wound healing rates at day 3 post-injury. Data are expressed as mean ± SD (n = 6). Statistical significance was assessed by one-way ANOVA with Tukey's post hoc test: ns, not significant.

**
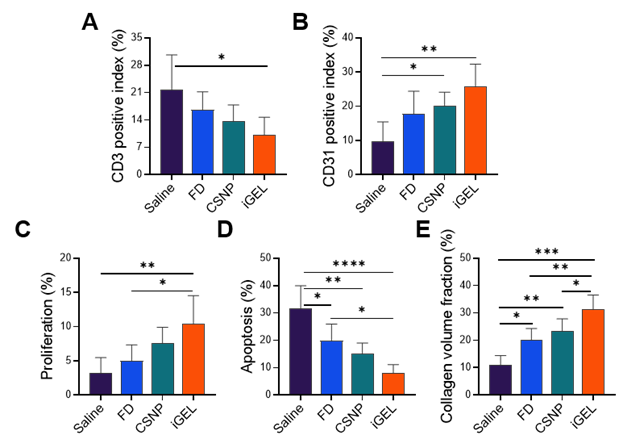
**

**Fig.S33.** Quantitative analysis of CD3 (A), CD31 (B), Cyclin D1 (C), TUNEL (D), and Masson (E) staining, quantified by ImageJ software. Data are expressed as mean ± SD (n = 5). Statistical significance was assessed by one-way ANOVA with Tukey's post hoc test: **p* < 0.05; ***p* < 0.01; ****p* < 0.001; and *****p* < 0.0001.

**
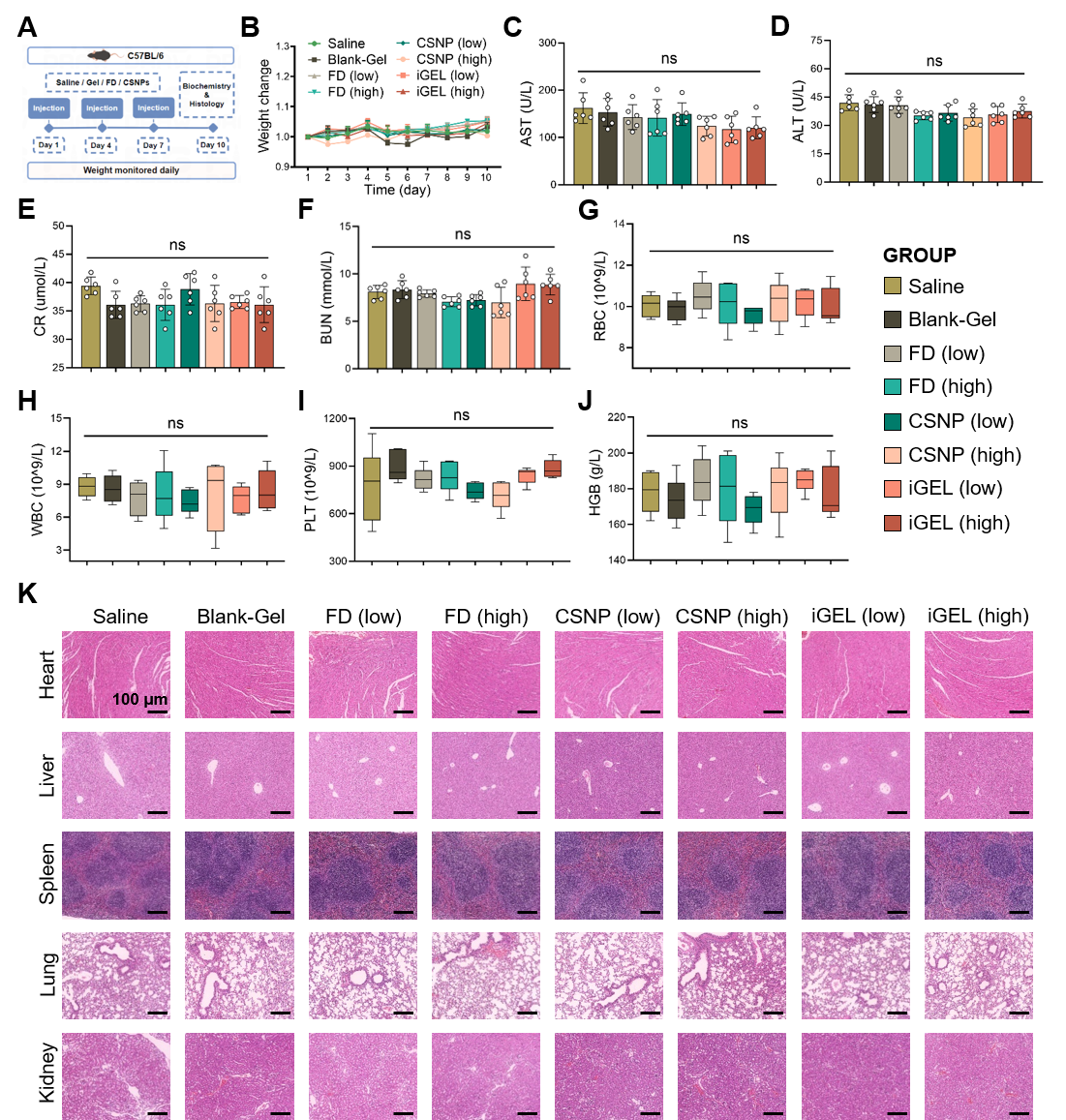
**

**Fig.S34. Safety evaluation of iGEL treatment.** (A) Schematic of the experimental design for safety evaluation. (B) Body weight changes during the treatment period. (C-F) Serum biochemical indices of hepatic and renal function. (G-J) Post-treatment changes in peripheral blood counts and hemoglobin levels. (K) Histopathological evaluation of major organs (heart, liver, spleen, lungs, and kidneys) by H&E staining. Scale bar: 100 μm. Data are expressed as mean ± SD (n = 6). Statistical significance was assessed by one-way ANOVA with Tukey's post hoc test: ns, not significant.


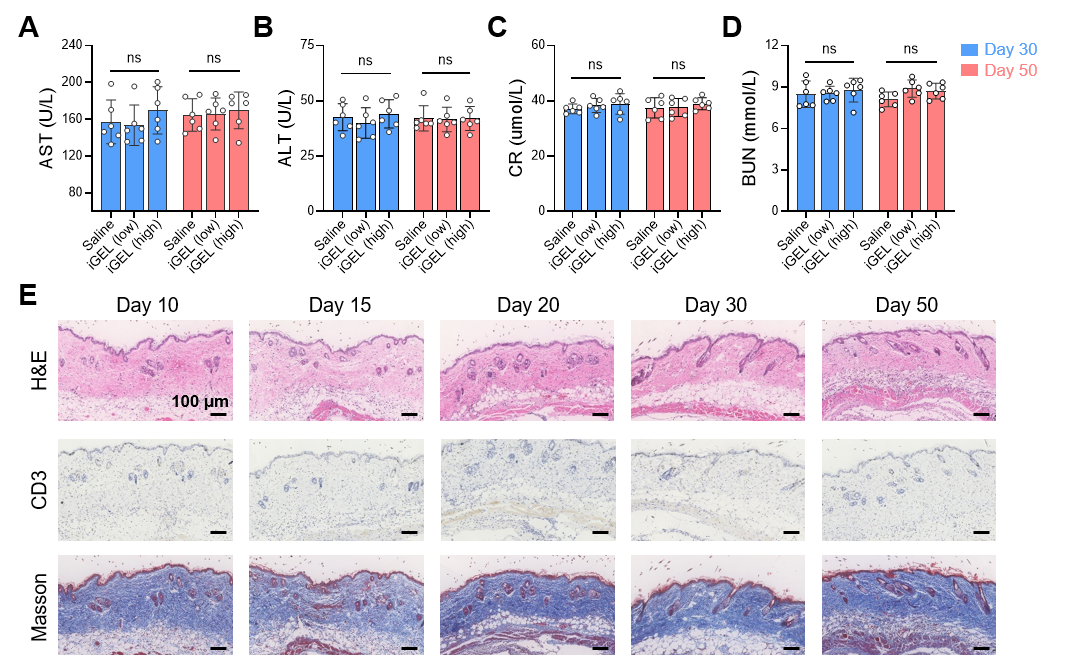


**Fig.S35. Long-term biocompatibility analysis of iGEL.** (A-D) Serum biochemical indices of hepatic and renal function on post-treatment days 30 and 50. Data are expressed as mean ± SD (n = 6). (E) Histopathological evaluation from post-treatment day 10 to day 50: H&E staining, immunohistochemistry (CD3^+^ T cells), and Masson's trichrome staining (blue: collagen). Scale bar: 100 μm. Statistical significance was assessed by one-way ANOVA with Tukey's post hoc test: ns, not significant.


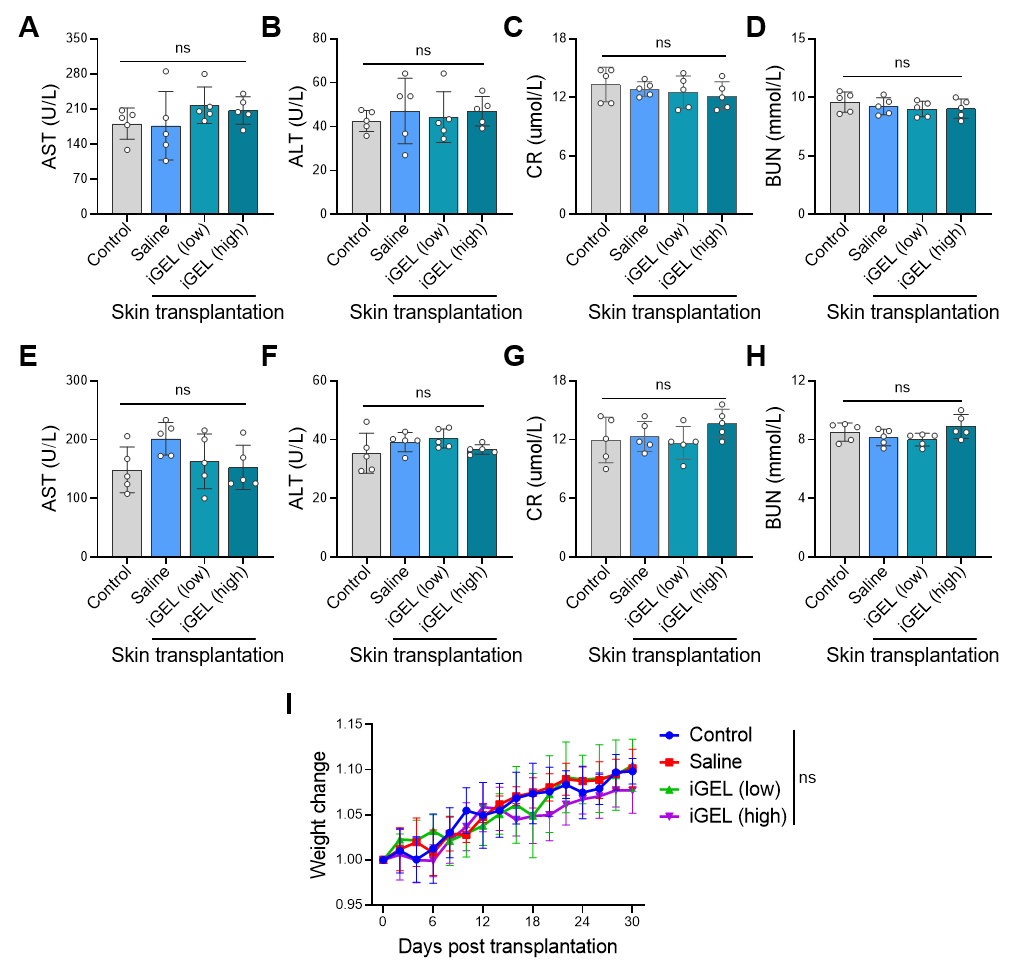


**Fig.S36. Assessment of liver and kidney function and body weight in allogeneic skin-transplanted mice treated with iGEL.** (A-D) Liver and kidney function markers measured on post-transplantation day 10, when allografts were present: (A) AST, (B) ALT, (C) CR, and (D) BUN. Data are shown for Control (healthy mice), Saline, iGEL low dose, and iGEL high dose groups. (E-H) Liver and kidney function markers measured on post-transplantation day 30, after allograft loss: (E) AST, (F) ALT, (G) CR, and (H) BUN. (I) Body weight of mice throughout the study period. Data are expressed as mean ± SD (n = 5). Statistical significance was assessed by one-way ANOVA with Tukey's post hoc test: ns, not significant.

**Table S1.** Primers used in this study for PCR.

| Gene | Forward primer | Reverse primer |
| --- | --- | --- |
| Cyclin D1 | 5' - GCGTACCCTGACACCAATCTC | 5' - CTCCTCTTCGCACTTCTGCTC |
| Cyclin A2 | 5' – GCCTTCACCATTCATGTGGAT | 5' – TTGCTCCGGGTAAAGAGACAG |
| PCNA | 5' - TTTGAGGCACGCCTGATCC | 5' - GGAGACGTGAGACGAGTCCAT |
| STAT3 | 5' - CAATACCATTGACCTGCCGAT | 5' - GAGCGACTCAAACTGCCCT |
| GAPDH | 5' - AGGTCGGTGTGAACGGATTTG | 5' - TGTAGACCATGTAGTTGAGGTCA |
| Fasn | 5' -GGAGGTGGTGATAGCCGGTAT | 5' -TGGGTAATCCATAGAGCCCAG |
| G0s2 | 5' -TAGTGAAGCTATACGTGCTGGGC | 5' -GTCTCAACTAGGCCGAGCA |
| Tmprss4 | 5' -CAACCCCTCAACAACCGTGAT | 5' -CTCAGCAGCACTGCAATGAT |
| Tnmd | 5' -ACACTTCTGGCCCGAGGTAT | 5' -GACTTCCAATGTTTCATCAGTGC |
| Serpine1 | 5' -TTCAGCCCTTGCTTGCCTC | 5' -ACACTTTTACTCCGAAGTCGGT |
| Cd209f | 5' -CTCTTTGGGCCTCTTTTTGCT | 5' -AGTATGCACGAATCCTGGAGA |
| Nlrp3 | 5' -ATTACCCGCCCGAGAAAGG | 5' -TCGCAGCAAAGATCCACACAG |
| Ptgs2 | 5' -TTCAACACACTCTATCACTGGC | 5' -AGAAGCGTTTGCGGTACTCAT |
| Lcn2 | 5' -TGGCCCTGAGTGTCATGTG | 5' -CTCTTGTAGCTCATAGATGGTGC |
| Nos2 | 5' -GTTCTCAGCCCAACAATACAAGA | 5' -GTGGACGGGTCGATGTCAC |
